# Therapeutic efficacy of human adipose mesenchymal stem cells in Crohn’s colon fibrosis is improved by IFN-γ and kynurenic acid priming through indoleamine 2,3-dioxygenase-1 signaling

**DOI:** 10.1186/s13287-022-03157-8

**Published:** 2022-09-08

**Authors:** Yixin Ye, Xiaomei Zhang, Dongsheng Su, Yushuang Ren, Fuyi Cheng, Yunqi Yao, Gang Shi, Yanhong Ji, Shuang Chen, Pengyi Shi, Lei Dai, Xiaolan Su, Hongxin Deng

**Affiliations:** State Key Laboratory of Biotherapy and Cancer Center/Collaborative Innovation Center of Biotherapy, West China Hospital, Sichuan University, Ke-yuan Road 4, No. 1, Gao-peng Street, Chengdu, 610041 Sichuan People’s Republic of China

**Keywords:** Human adipose tissue-derived MSCs, Pretreated hADSCs with IFN-γ and KYNA, Crohn’s disease colonic fibrosis rat model, Indoleamine 2,3-dioxygenase-1

## Abstract

**Background:**

Inflammatory bowel diseases (IBD) are chronic relapsing–remitting inflammatory diseases of the gastrointestinal tract that are typically categorized into two subtypes: Crohn's disease (CD) and ulcerative colitis (UC). Although MSCs therapy has achieved encouraging outcomes in IBD therapy, objective responses are limited in colon fibrosis stenosis owing to the complicated microenvironment of CD and MSCs heterogeneity of quality. Here, we chose IFN-γ and kynurenic acid (KYNA) to overcome the low response and heterogeneity of human adipose-derived MSCs (hADSCs) to treat IBD and expand the therapeutic effects based on the excellent ability of IFN-γ and KYNA to promote indoleamine 2,3-dioxygenase-1 (IDO-1) signaling, providing a potential protocol to treat IBD and fibrosis disease.

**Methods:**

hADSCs were isolated, cultured, and identified from human abdominal adipose tissue. The CD pathology-like acute colitis and chronic colon fibrosis rat model was induced by 2,4,6-trinitrobenzen sulfonic acid (TNBS). hADSCs were pretreated in vitro with IFN-γ and KYNA and then were transplanted intravenously at day 1 and 3 of TNBS administration in colitis along with at day 1, 15, and 29 of TNBS administration in chronic colonic fibrosis. Therapeutic efficacy was evaluated by body weights, disease activity index, pathological staining, real-time PCR, Western blot, and flow cytometry. For knockout of IDO-1, hADSCs were transfected with IDO-1-targeting small gRNA carried on a CRISPR-Cas9-lentivirus vector.

**Results:**

hADSCs treated with IFN-γ and KYNA significantly upregulated the expression and secretion of IDO-1, which has effectively ameliorated CD pathology-like colitis injury and fibrosis. Notably, the ability of hADSCs with IDO-1 knockout to treat colitis was significantly impaired and diminished the protective effects of the primed hADSCs with IFN-γ and KYNA.

**Conclusion:**

Inflammatory cytokines IFN-γ- and KYNA-treated hADSCs more effectively alleviate TNBS-induced colitis and colonic fibrosis through an IDO-1-dependent manner. Primed hADSCs are a promising new strategy to improve the therapeutic efficacy of MSCs and worth further research.

**Supplementary Information:**

The online version contains supplementary material available at 10.1186/s13287-022-03157-8.

## Background

Inflammatory bowel disease (IBD) includes Crohn's disease (CD) and ulcerative colitis (UC) [1], the etiology of which remains unclear. At present, it is believed that individuals carrying susceptible genes have abnormal bowel immune responses under the stimulation of various environmental factors, which are characterized as chronic and recurrent [2]. Currently, the step-up/step-down therapy for CD includes 5-ASA or sulfasalazine, glucocorticoids, azathioprine, anti-TNF antibody, and surgery, although a variety of combination plans can alleviate intestinal inflammation in the active stage in a short period of time. However, for repeated inflammation and fibrosis control is limited. Long-term chronic inflammation stimulates the activation of a variety of interstitial cells, including myofibroblasts, and produces a large amount of extracellular matrix (ECM) precipitation, which leads to fibrosis of the intestinal wall and intestinal stenosis [3]. Therefore, there is an urgent clinical need for new strategies to prevent or control the progression of intestinal fibrosis.

Mesenchymal stem cells (MSCs) are a group of heterogeneous stem cells that exist in nearly all tissues and can differentiate into specific types of cells [4]. Like other tissue-derived MSCs, human adipose-derived MSCs (hADSCs) have low immunogenicity and can be autografted to avoid ethical issues related to clinical application, making them ideal cell therapy seed cells [5]. MSCs are considered promising candidates for the treatment of IBD due to their potential immunomodulatory and tissue repair function [6]. Many studies have suggested that MSCs can modulate the biological activity of a variety of immune cells, such as T cells, B cells, natural killer cells, macrophages, and neutrophils. Although the immunomodulatory mechanism of MSCs is still not fully elucidated, it is generally believed that MSCs have an immunosuppressive role mainly through cell-to-cell contact and secretion of soluble factors, such as insulin-like growth factor-1, nitric oxide, prostaglandin E2, transforming growth factor-β1 (TGF-β1), hepatocyte growth factor, cyclooxygenase-2 (COX-2), and indoleamine 2,3-dioxygenase-1 (IDO-1) [7, 8]. Studies have shown that the immunosuppressive ability of MSCs depends on inflammatory environmental stimuli, such as IFN-γ and TNF-α [9]. Therefore, the heterogeneity of the immunosuppressive ability and mechanism of MSCs mainly depends on the tissue type and microenvironment in which they are located. Studies have demonstrated an integral role of IDO-1 in the immunomodulatory capacity of MSCs [10]. This enzyme catalyzes the first and rate-limiting step of tryptophan catabolism along the kynurenine pathway. Notably, IDO-1 and tryptophan metabolites, such as kynurenine, kynurenic acid (KYNA), and 3-hydroxyanthranilic acid, have been documented to modulate the functions of immune cells and regulate the expression of inflammation-associated genes. IDO-1 is expressed at low levels under normal conditions. A variety of inflammatory cytokines, especially IFN-γ, can induce the expression of IDO-1. Studies have found that KYNA can also induce the expression of IDO-1 in vitro [11, 12]. However, little is known about the role of IDO-1 in the regulation of the MSC-based innate immune response, especially in vivo.

In the present study, we established a trinitrobenzene sulfonic acid (TNBS)-induced chronic colonic fibrosis rat model characterized by CD pathology-like features and found that IDO-1 is necessary to achieve the effect of hADSCs-based treatment in an IBD model. We demonstrated that IFN-γ- and KYNA-pretreated hADSCs increased IDO-1 and IL-10 expression, promoted M2 macrophage polarization, alleviated inflammation, stimulated epithelial regeneration, decreased ECM accumulation, inhibited epithelial–mesenchymal transition (EMT) progression, and had better therapeutic efficacy in a rat IBD colonic fibrosis model than non-pretreated hADSCs.

## Materials and methods

### hADSCs culture and preparation

Adipose tissue was provided by the West China Hospital of Sichuan University and was taken from a small amount of residual abdominal subcutaneous fat tissue after skin transplantation and repair in healthy men. Tissue sample collection was approved by the Medical Ethics Committee of Sichuan University (K2018109-1).

hADSCs were isolated, amplified, and characterized, as previously reported [13]. Briefly, the adipose tissue was repeatedly cleaned with sterile PBS, torn with tweezers, and placed in a 50-mL centrifuge tube. Type I collagenase was added to the tube at a final concentration of 0.1%, which was then placed in a 37 °C shaker and digested for approximately 30 min. The filtrate was then collected and centrifuged at 1000 rpm for 3 min. The supernatant was discarded, and the cell pellet was washed twice with 30 mL basal medium (MSCBM), after which was resuspended in a complete medium (95% MSCBM with 5% EliteGeo™-Advanced). The suspension was then inoculated in a Petri dish and placed in a 37 °C incubator with 5% CO_2_ for culturing. P5 cells were used in this study, and phenotype markers along with tri-lineage differentiation were examined before animal experiments. All details regarding the characterization of the cultured hADSCs are shown in Additional file 1: Fig. S1.

### Transfection of hADSCs with IDO-1 sgRNA

For IDO-1 knockout, hADSCs were transfected with IDO-1-targeting sgRNA carried on a CRISPR-Cas9-lentivirus vector and sequenced with the primer hU6-F to obtain positive transformants (Additional file 1: Fig. 5). The lentiviral vector (Plasmid #52,961; Addgene, Watertown, MA, USA) was a gift from Feng Zhang Laboratory, MIT. Lentiviral sgRNA was generated by co-transfection of the Trans-Lentiviral Packaging Mix with the IDO-1 sgRNA transfer vector into HEK 293 T packaging cells. Supernatants containing lentivirus expressing IDO-1 sgRNA were harvested 48 h after transfection. The lentiviruses were purified by ultra-centrifugation (70,000 × g at 4 °C for 2 h), and the lentivirus titers were determined. The IDO-1 sgRNA sequences are listed in Additional file 1: Table S1. hADSCs were incubated with lentivirus and polybrene (5 μg) for 24 h. Puromycin (10 µg; Gibco, Waltham, MA, USA) was added to the culture medium to select the transfected cells.

The IDO-1 knockout efficiency was verified by Western blotting (Additional file 1: Fig. 5).

### Transcriptome sequencing

From three donors, P5 hADSCs and IFN-γ- and KYNA-pretreated hADSCs cells were digested and counted, after which 1 mL TRIzol reagent was added to 3–4 × 10 [6] cells. All of the liquid was transferred to RNase-free tubes and stored at −80 °C until later use. Total RNA was sent to Shanghai Majorbio Bio-Pharm Technology Co., Ltd., for sequencing. The data were analyzed on the free online Majorbio Cloud Platform (www.majorbio.com).

### Animal experiments and experimental design

Male Wistar rats were purchased from Beijing Huafukang Biotechnology Co., Ltd. (China) and were 8 weeks old (200 ± 20 g). The experimental animal center of the State Key Laboratory of Biotherapy was used to conduct animal experiments in strict accordance with specific pathogen-free conditions.

TNBS-induced colitis methods were followed as shown previously [14]. Briefly, rats were anesthetized by intramuscular injection of Zoletil®50 (zolazepam–tiletamine) at a dose of 100 μL and were treated with 1 mL 2% TNBS/ethanol solution (preparation method is shown in Additional file 1: Table S2) infused slowly into the rectum through a catheter equipped with a 1-mL syringe and held in a vertical position for 3 min to ensure distribution throughout the colon.

In the acute TNBS-colitis rat transplant experiment, 8-week-old male Wistar rats were randomly allocated into one of four groups: normal saline (control group), TNBS only with PBS (PBS group), TNBS with untreated hADSCs (hADSCs group), and TNBS with hADSCs pretreated with IFN-γ and KYNA (I + K-hADSCs group). Untreated hADSCs or primed hADSCs (1.5 × 10 [6] cells) in 1 mL PBS were injected on days 1 and 3 through the rat tail vein. The body weight and DAI score of each rat were recorded daily according to standard protocols [15]. On day 7, we observed the progress of colitis through colonoscopy in vivo and recorded statistical inflammation scores [16]. Rats were killed on day 10, and samples, including peripheral blood, colon tissue, spleen, and mLN, were harvested.

In the chronic colon fibrosis, TNBS-induced colitis rat transplant experiment repeated injections into the rectum of TNBS/ethanol solution (1.0%, 1.0%, 1.5%, 1.5%, 2%, and 2%, Additional file 1: Table S2) once a week for 6 weeks. The changes in body weight and DAI scores were evaluated daily. Wistar rats were randomly allocated into one of four groups: control, PBS, hADSCs, and I + K-hADSCs. Non-pretreated hADSCs or primed hADSCs (2.0 × 10^6^ cells) were administered intravenously on days 1, 15, and 29. Rats were killed on day 46, and peripheral blood, spleen, mLN, and colon samples were harvested on days 7 and 40 to observe the progress of colitis through colonoscopy in vivo and statistical scores were recorded.

For the hADSCs-IDO-1 knockout transplant experiment, acute colitis was induced using TNBS, and then primed hADSCs^IDO−1−KO^ or primed hADSCs (1.5 × 10^6^ cells) were administered intravenously on days 1 and 3. Colonoscopy and statistical inflammation scores were performed on day 7, rats were killed on day 10, and colon samples were harvested.

Experimental procedures involving animals were approved by the Sichuan University Medical Ethics Committee (K2018109-2).

### MPO activity assay

Measurement of MPO activity was taken to monitor neutrophil infiltration using an MPO kit (Jiancheng, Nanjing, China).

### Histopathology and immunofluorescence staining

A 5-μm frozen section of colon tissues was prepared from each group and stained with H & E. For immunohistochemistry, sections were blocked with 5% bovine serum albumin (BSA) for 45 min at 37 °C. The sections were incubated with primary antibodies (1:200 in PBS) at 4 °C overnight incubation. For fluorescence staining, the processing of cells was the same as that for tissue processing. Nuclei were counterstained with DAPI (Invitrogen, Waltham, MA, USA). Antibody staining was performed using a fluorescence microscope (Olympus). Detailed information on the antibodies used is shown in Additional file 1: Table S3.

### Blood routine analysis

Rats were anesthetized, and the peripheral blood of each group was collected through the inferior vena cava, which was sent to WestChina-Frontier PharmaTech Co., Ltd., for routine blood testing. The test included white blood cells (WBC), neutrophils (NEUT), lymphocytes (LYMPH), and monocytes (MONO) to evaluate the level of inflammation in each group of rats.

### In vivo tracking imaging

For the Dil or DiR labeling of hADSCs or pretreated hADSCs, cells (3.0 × 10^6^) were incubated with 5 μL/mL CM-DiI (1 mg/mL; C7001, Invitrogen) or 10 μL/mL DiR’ (10 μM; 12,731, Invitrogen) at 37 °C for 20 min and 4 °C for 40 min, and the labeling efficiency was measured by fluorescence microscopy (Additional file 1: Fig. 6).

### Flow cytometry

Fluorochrome-conjugated monoclonal antibodies against rat or human molecules for flow cytometry staining (antibody clone, fluorochrome, supplier, and catalog number) are listed in Additional file 1: Table S3. Fixable viability stain 620 (FVS-620, 564,996, BD Biosciences) was used to discriminate between live and dead cells, which were then blocked with Fc-block (553,142, BD Biosciences, Franklin Lakes, NJ, USA) and stained with antibodies. Intracellular cytokine (CD206, Proteintech, Rosemont, IL, USA) staining was performed using a Fixation/Permeabilization Kit (BD Biosciences). The data were acquired using a NovoCyte Flow Cytometer.

### Western blot analysis

Cells or tissues were lysed in a mammalian protein extraction reagent (Pierce, Rockford, IL, USA). Proteins were separated by SDS-PAGE and transferred onto PVDF membranes (Bio-Rad, Tokyo, Japan). Blots were saturated with 5% skimmed milk in TBS-T for 1 h at room temperature, followed by overnight incubation with anti-α-SMA (Abcam, Cambridge, UK), anti-vimentin (CST, Danvers, MA, USA), anti-laminin rabbit mAb (Abcam), anti-IDO rabbit antihuman antibody (Proteintech), anti-iNOS rabbit polyclonal antibody (Proteintech), and anti-COX-2 rabbit polyclonal antibody (Proteintech). After washing with TBS-T, the membranes were incubated with sheep anti-rabbit or anti-mouse IgG-HRP-conjugated intact antibodies (GE Healthcare Bio-Sciences KK). Monoclonal antibodies against human GAPDH were used as controls for protein loading (sc-32,233, Santa Cruz Biotechnology). Detailed information on the Western blotting antibodies is provided in Additional file 1: Table S3.

### qPCR

Total RNA was extracted using TRIzol reagent (Invitrogen, USA) and quantified using a spectrophotometer. qPCR was performed using a Step-One Real-Time PCR system (Takara), according to the manufacturer’s instructions, with the CFX96 Touch™ Real-Time PCR Detection System (Bio-Rad Life Science Research). The quantity of mRNA was calculated using the 2^−△△Ct^ method, and the gene expression was normalized to β-actin. All reactions were performed in triplicate, and the primer sequences are listed in Additional file 1: Tables S4 and S5. Primers used for the experiment were synthesized by Chengdu Tsingke Biotechnology Co., Ltd.

### Statistical analyses

Experimental data were analyzed using GraphPad Prism software (version 8.0). All values are expressed as mean ± standard error of the mean (SEM). Two-tailed unpaired *t* tests were used for comparisons between two groups. Comparisons among the groups were performed using one-way analysis of variance (ANOVA) with Tukey’s multiple comparison or two-way ANOVA with Tukey’s multiple post-comparison. Statistical significance was set at *p* < 0.05.

## Results

### Characterization of IFN-γ- and KYNA-primed hADSCs

High-purity hADSCs with a distinct fibroblastic morphology were isolated from human adipose tissue (Additional file 1: Fig. S1a). As determined by flow cytometry analysis of the specific cellular markers, the cells obtained expressed positive markers CD105, CD90, CD73, CD44, and CD29 (more than 99%), while negative markers lacked CD45, CD34, CD14, CD11b, and HLA‐DR (less than 0.5%), consistent with the characteristic surface markers expressed on MSCs (Additional file 1: Fig. S1b). We identified the multipotency of these cells by adipogenic, osteogenic, and chondrogenic differentiation in differentiation medium. The adipogenic, osteogenic, and chondrogenic abilities of these cells were identified by staining for neutral lipids, calcium nodules, and cartilage, respectively, using Oil Red O, Alizarin Red, and Alcian Blue. The three-line differentiation staining results indicated that adipogenesis, osteogenesis, and chondrogenesis were successfully induced (Additional file 1: Fig. S1c).

Next, we wanted to clarify whether the combination of IFN-γ- and KYNA-primed hADSCs (I + K-hADSCs) affected their characteristics. The hADSCs pretreated with IFN-γ (20 ng/mL) and KYNA (50 μM) for 24 h in vitro showed no obvious changes in cell morphology (Fig. S1d), and the detected surface markers did not have a significant impact (Fig. S1e), indicating that stimulation of hADSCs with IFN-γ and KYNA does not change their identity at this dose.

### Transcriptome analysis of IFN-γ- and KYNA-primed hADSCs

**Fig. 1:**
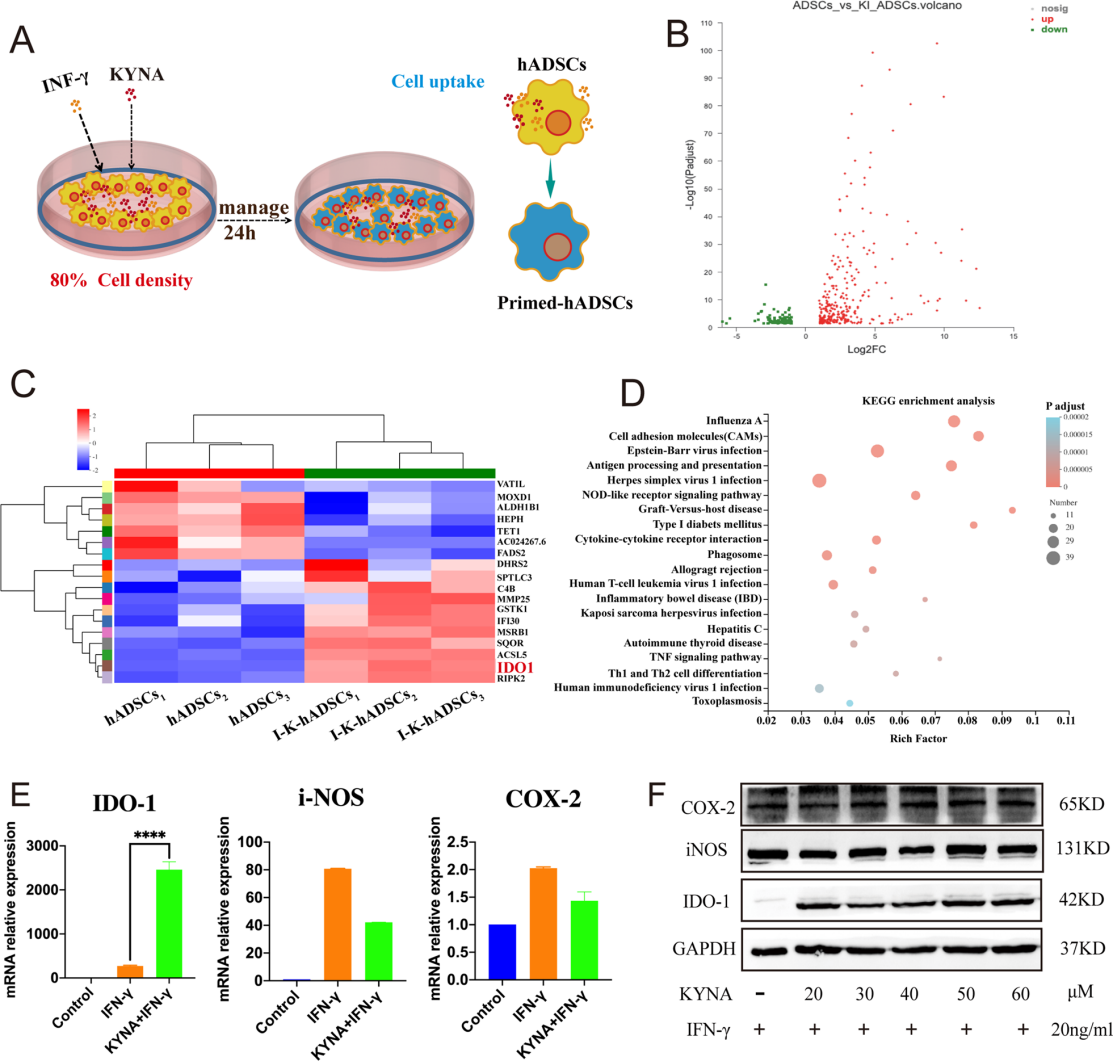
IDO-1 of hADSCs induced by pretreated IFN-γ combined with KYNA. (**A**) IFN-γ (20 ng/mL) combined with KYNA (50 μM) stimulated P5 hADSCs pattern. (**B**) Volcano plot of P5 I + K-hADSCs and hADSCs transcriptome sequencing. (**C**) Cluster analysis of hADSCs and IFN-γ and I + K-hADSCs. (**D**) KEGG enrichment analysis of hADSCs and I + K-hADSCs. (**E**) qPCR results of each group treated or untreated for 24 h, mRNA expression levels of IDO-1, iNOS and COX-2 in Control group (P5 hADSCs), IFN-γ group (IFN-γ 20 ng/ml treated P5 hADSCs), KYNA + IFN-γ group (IFN-γ 20 ng/ml, KYNA 50 μM treated P5 hADSCs). (**F**) Western blot detected the hADSCs and I + K-hADSCs protein expression levels of IDO-1, iNOS, and COX-2. Data are represented as mean ± SEM

To clarify the influence of IFN-γ and KYNA on the immune regulation ability of hADSCs, we sequenced the cell samples from the hADSCs and primed hADSCs groups (Fig. 1a). We performed a cluster analysis of the differentially expressed genes (DEGs) (Additional file 1: Fig. S1f). The in vitro primed hADSCs and hADSCs groups showed significant differences in gene expression profiles. Gene Ontology enrichment analysis showed that the DEGs were enriched in 21 biological process-related pathways, 15 cellular component-related pathways, and 11 molecular function-related pathways. We displayed the top 20 significantly enriched signal pathways in the gene ontology analysis results through bubble diagrams (Additional file 1: Fig. S1g). The results of pathway enrichment analysis showed that compared with hADSCs in the basic state, the DEGs produced after IFN-γ, KYNA, and hADSCs co-culture were mainly enriched in signals related to antigen processing and presentation, cell adhesion molecule pathways, cytokine and receptor interaction, Th1, Th2, and Th17 cell differentiation, IBD, IFN-γ signaling pathways, TNF signaling, and anti-viral infection signals in the pathway. We analyzed the DEGs of the hADSCs and primed groups, *p*-adjust < 0.05, up/down difference multiple of 2 (which is considered to be a significant difference), and the expression difference volcano map (Fig. 1b). A total difference of 408 genes were found which included 298 upregulated and 110 downregulated genes. The DEGs were further analyzed (Fig. 1c) where the key differential gene molecule IDO-1 in the primed group was screened out and its expression was significantly increased in which the three different donors showed a trend of consistent change. Kyoto Encyclopedia of Genes and Genomes analysis demonstrated that the primed group was enriched in pathways associated with immune response, especially antigen processing and presentation (Fig. 1d).

We further confirmed via qPCR and Western blotting that IFN-γ combined with KYNA treatment of hADSCs compared to untreated hADSCs effectively upregulated the expression of IDO-1, and the expression of inducible nitric oxide synthase (iNOS) and COX-2 was increased in the primed group, but the increase was not significant (Fig. 1e and f). Therefore, we demonstrated that in vitro IFN-γ and KYNA combined pretreatment of hADSCs can effectively upregulate IDO-1 gene and protein expression levels.

### IFN-γ- and KYNA-primed hADSCs were more effective than untreated hADSCs to alleviate TNBS-induced colitis and colonic fibrosis

**Fig. 2:**
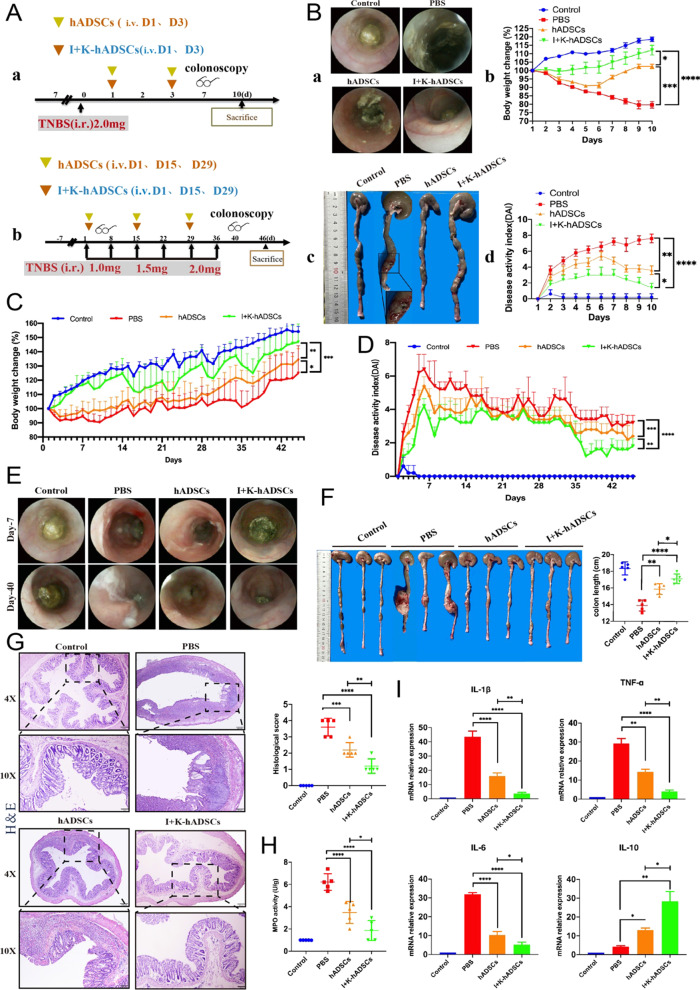
The primed hADSCs were more effective than untreated hADSCs to alleviated TNBS-induced colitis and colonic fibrosis. (**A**) Experimental layout of primed and untreated hADSCs treatment in the acute colitis and chronic colon fibrosis; (**B**) Therapeutic efficacy evaluation of acute TNBS model. a. endoscopy observed on the 7th day. b. body weight change percentage (*n* = 5). c. anatomic observed on the 10th day. d. DAI scores (*n* = 5); (**C**) body weight change percentage (*n* = 5); (**D**) DAI scores (*n* = 5); (**E**) endoscopy observed on the 7th and 40th day; (**F**) rats were killed at the 7th week, the length of the colon was counted and the lesions of the colon were observed; (**G**) H&E staining was performed to observe the structural integrity of intestinal and the infiltration of immune cells in each group and score (Magnification 4X and 10X; Scale bar = 200, 100 μm; *n* = 5); (**H**) MPO activity detected (*n* = 5); (**I**) qPCR detection mRNA expression changes of inflammatory cytokines IL-1β, TNF-α, IL-6 and anti-inflammatory molecule IL-10 (*n* = 5); *p < 0.05, **p < 0.01, ***p < 0.001, ****p < 0.0001. Data are represented as mean ± SEM

To identify the immunomodulatory and antifibrotic effects of IFN-γ- and KYNA-primed hADSCs, we used a rat model of TNBS-induced colitis and fibrosis (Fig. 2a). First, an acute TNBS-induced colitis model was induced, and primed hADSCs and untreated hADSCs were injected intravenously. Compared to the control, PBS, and hADSCs groups, the primed group colonoscopy observation on day 7 found that the primed group’s intestinal mucosa was normal and that there was slight congestion. Since then, we counted the endoscopic colitis score, including intestinal thickening, colon wall bloody, visible intestinal fiber deposition, intestinal mucosal surface granularity, and fecal viscosity, and found that compared to the other groups, the primed group enteritis index score was the lowest (Fig. 2b and Additional file 1: Fig. S2a). To further evaluate the treatment effectiveness, we discovered that the primed group exhibited reduced body weight loss and had lower disease activity index (DAI, including weight change, bloody diarrhea, and lassitude) scores (Fig. 2b). Additionally, after killing the rats by euthanasia, the colon tissue was collected for observation and the macroscopic damage score was evaluated; the primed hADSCs treatment reduced colon shortening, which is one of the hallmarks of colitis (Additional file 1: Fig. S2b). Interestingly, we found that the TNBS-induced rat colitis model exhibited obvious transmural colon damage (Fig. 2b), which is a pathological feature of clinical Crohn’s disease. We also investigated myeloperoxidase (MPO) activity in the damaged colon and found that it was significantly decreased in the primed hADSCs group (Additional file 1: Fig. S2c).

Total RNA was extracted from colon tissues, and the mRNA levels of the target genes were quantified by RT-qPCR. Among the measured inflammatory cytokines, IL-1β, CXCL1, IL-6, and TNF-α were significantly reduced, and the anti-inflammatory cytokine IL-10 was significantly increased in the primed hADSCs group (Fig. S2d). Pathologic examination confirmed the improvement in TNBS-induced colitis symptoms, which were caused by primed hADSCs. H&E sections revealed that the typical pathological manifestations of TNBS-induced colitis (epithelial destruction, crypt loss, and massive inflammatory cell infiltration) were significantly ameliorated by the non-pretreated hADSCs and further still by the primed hADSCs (Additional file 1: Fig. S2e).

Chronic TNBS colitis and a colon fibrosis model were induced, and hADSCs and primed hADSCs were injected intravenously (Fig. 2a). Similar to the acute colitis model, body weight loss was reduced in the primed group, which also had lower DAI scores and MPO activity (Fig. 2c, d and h). Colonoscopy revealed that the TNBS-induced colon fibrosis group had obvious Crohn’s disease pathologic changes, including intestinal wall edema, enteric inflammatory exudate, and serious lumen stenosis; the hADSCs group showed slight congestion that was further ameliorated by the primed hADSCs (Fig. 2e and Additional file 1: Fig. S2f).Additionally, anatomical analysis of colon fibrosis rats after dissection showed that the colon was shortened, twisted, stiff, and deformed, which adhered to the surrounding tissues. Furthermore, penetrating ulcers with stool retention in the obvious area of colon dilation were observed and the lesion was covered with a pseudo-membrane (Additional file 1: Fig. S2g). The average colon length was reduced to a lesser extent in the primed group (Fig. 2f and Additional file 1: Fig. S2h). Significantly, H&E sections showed that the colorectal wall was markedly thicker in the PBS group, with excessive granulation and hypertrophy of the muscularis propria; however, the colonic tissue in the primed hADSCs group had less erosion and lymphocytic infiltration in the epithelial and submucosal layers (Fig. 2g). The histological scores were also decreased in the untreated hADSCs and primed hADSCs groups, but the latter was more remarkable.

Immunofluorescence staining showed that many CD45^+^ cells infiltrated the lesions in the upper colon in the PBS group (Additional file 1: Fig. S3a). The regenerative capacity of the colonic mucosa is a key factor in the defense against injury and inflammation caused by various pathogenic factors. The integrity of crypt cells and epithelial mucosal barriers are essential elements for maintaining the proliferation of the epithelium [1]. The epithelial crypts in the primed hADSCs group exhibited greater proliferative potential, characterized by an increase in Ki-67^+^ cells, compared to the hADSCs and PBS control groups (Fig. S3b). Among the measured inflammatory cytokines, IL-1β, TNF-α, and IL-6 were significantly reduced, and the anti-inflammatory cytokine IL-10 was significantly increased in the primed hADSCs group (Fig. 2i). These results indicate the anti-inflammatory and anti-fibrosis effects of the primed hADSCs compared to the untreated hADSCs.

### IFN-γ- and KYNA-primed hADSCs significantly inhibited ECM deposition and EMT process

**Fig. 3:**
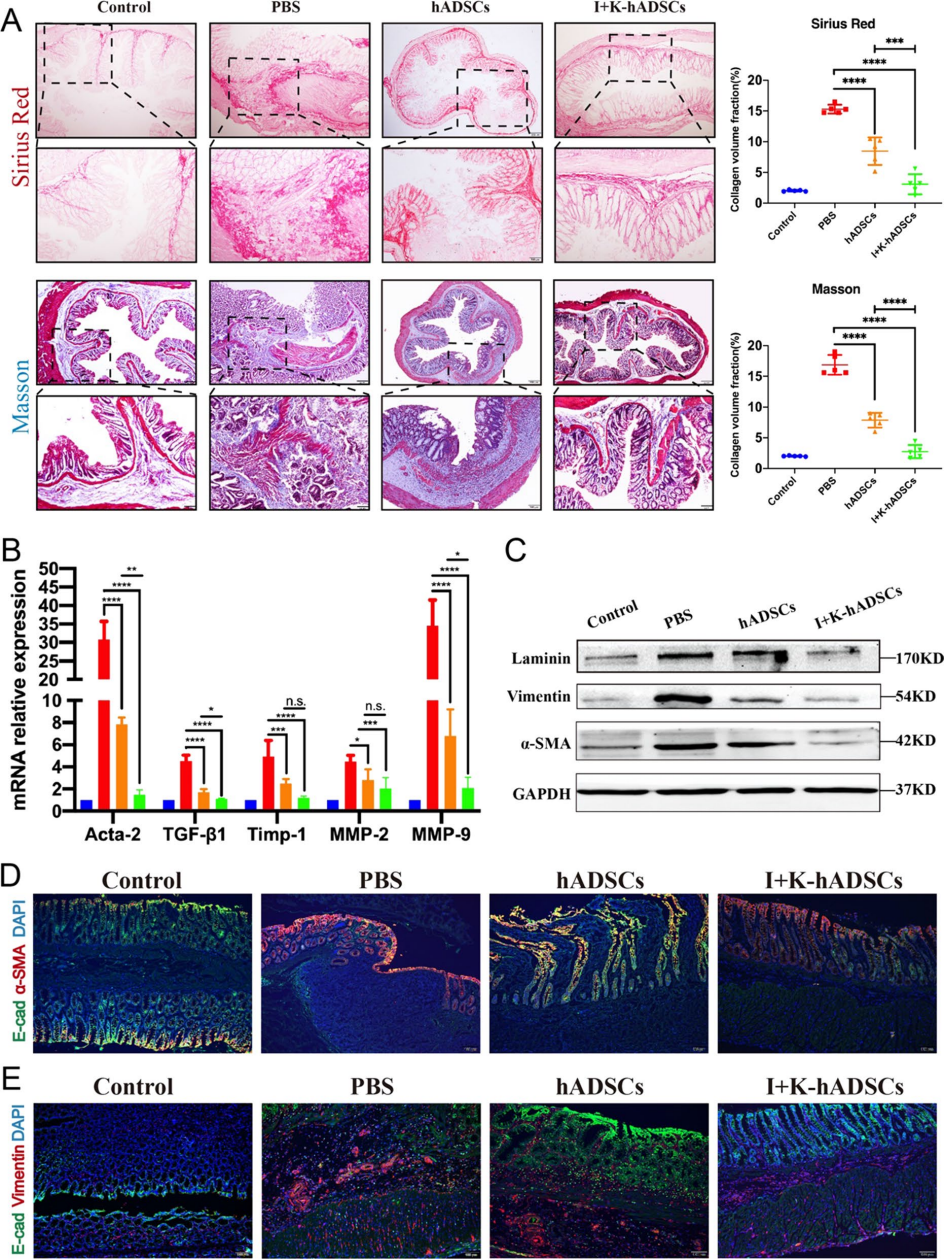
The IFN-γ- and KYNA-primed hADSCs significantly inhibited ECM deposition and EMT process. (**A**) Collagen staining analysis for therapeutic efficacy evaluation of colon fibrosis in rats; Up: Sirius Red staining; Bottom: Masson’s trichrome staining and Staining area statistics by the ImageJ software (*n* = 5); (**B**) qPCR analysis of fibrosis-related gene expression (Acta-2, TGF-β1, Timp-1, MMP-2, and MMP-9) in the colon on 46th day after treatment (*n* = 5); (**C**) Western blot analysis of laminin, vimentin, and α-SMA protein expression; (**D**) Immunofluorescence staining of the epithelial marker E-cad and myofibroblasts marker α-SMA in frozen sections of the colon (Scale bar, 100 μm); (**E**) Immunofluorescence staining of the epithelial marker E-cad and stromal marker vimentin in frozen sections of the colon (Scale bar, 100 μm); Data are represented as mean ± SEM

In TNBS-induced colitis rats, a large amount of ECM deposition is produced due to long-term inflammation of the colon tissue damage and re-repair process, leading to excessive proliferation and colon mesenchymal cell growth. The excessive accumulation and degradation of the ECM deficiency cause the formation of intestinal wall fibrosis and abnormal contraction of the ECM leading to the formation of intestinal obstruction [2]. We performed histological analysis of the colorectum removed on day 46. First, we analyzed collagen deposition in the fibrotic bowel using Sirius Red staining and observed obvious thickening of the muscularis propria, submucosa, and serosa layer, as well as a large number of collagen fiber depositions, with an increase in collagen fibers at the margin and inside of the injured zones in the TNBS-induced group compared with the control group (Fig. 3a). Masson’s trichrome staining demonstrated that continuous TNBS treatment caused severe fibrotic changes in the PBS group, and the fiber accumulation extended throughout the muscularis propria but was reduced and limited to the submucosa in the hADSCs group (Fig. 3a). Meanwhile, volume fraction statistics of Sirius Red and Masson staining of collagen fibrosis were conducted using the grayscale recognition software ImageJ (Fig. 3a, right). After hADSCs and primed hADSCs treatment, collagen fiber deposition improved significantly. Overall, these results suggest that the two types of pathological staining significantly decreased after hADSCs treatment and further still by the primed hADSCs in similar zones.

In order to further investigate the ECM remodeling process induced by TNBS after 6 weeks, matrix metalloproteinase (MMP), tissue inhibitor of metalloproteinase (TIMP), collagen, and α-smooth muscle actin (α-SMA) gene expression were studied according to Hodson et al. [17]. TGF-β1, as a multipotent cytokine, can inhibit inflammation and promote the formation of fibrosis and overexpression promotes the progression of intestinal fibrosis [18]. We observed that the relative levels of Acta-2, TGF-β1, Mmp-2, and Mmp-9, as well as their inhibitors Timp-1, were increased significantly in the colonic mucosal TNBS-induced colon tissue compared with the controls (Fig. 3b). This result highlights the active process of ECM remodeling with a balance toward fibrogenesis in injured tissues. Interestingly, primed hADSCs transplantation decreased the mRNA levels of enzymes involved in ECM remodeling, which was associated with a decrease in the relative levels of Acta-2 and TGF-β1 expression (Fig. 3b). Additionally, we found that many collagen IV-positive cells were observed in the intestinal lesions of the PBS group, but not in the hADSCs and primed hADSCs groups (Additional file 1: Fig. S3c). There was only a small amount of laminin in the normal intestinal stroma and the increase in myofibroblasts during fibrosis led to the synthesis and secretion of collagen and laminin [19]. Western blotting and immunofluorescence of colon proteins showed decreased expression of α-SMA and laminin in the primed hADSCs group compared to the untreated hADSCs and PBS groups (Fig. 3c, d and Additional file 1: Fig. S3d).

EMT refers to the loss of polarity of epithelial cells, transformation into fibroblast morphology, reduced contact with surrounding cells, and increased migration and mobility. Previous studies have shown that EMT processes also play a dual role, similar to TGF-β1 in physiology. Physiological conditions are involved in tissue repair and structural recovery, and the continuous activation of this process leads to tissue fibrosis [20]. During EMT, the significant change was the disappearance of epithelial markers in which the expression of E-cadherin was significantly reduced. The main function of E-cadherin is to maintain the lateral contact of epithelial cells, cell contact, and relative immobility by adherent junctions. Vimentin is a cytoskeletal protein that can reduce the transport of E-cadherin to the cell surface and is the most common marker of mesenchymal cells and a major molecular marker of EMT [21]. Western blotting and immunofluorescence revealed that the proportion of epithelial marker E-cadherin-positive cells in the PBS group was significantly lower than that in the control group, and the epithelial structure was obviously injured (Fig. 3d and e). Many α-SMA-positive myofibroblasts filled the epithelial cell zone, indicating that the fibrosis process was still progressing in the PBS group (Fig. 3d). Meanwhile, vimentin-positive fibroblasts also appeared in large numbers in the serosal muscle layer, E-cadherin-labeled epithelial cells were recovered after hADSCs transplantation, and the intestinal structure of the primed hADSCs group was clearer and more complete (Fig. 3c and e). The proportion of α-SMA- and vimentin-positive fibroblasts and stromal cells in the primed hADSCs group was significantly lower than that in the PBS and hADSCs groups. Taken together, these results show that primed hADSCs can effectively inhibit ECM deposition and the EMT process, thus maintaining the stability of the intestinal structure compared to untreated hADSCs.

### IFN-γ- and KYNA-primed hADSCs promoted polarization of M2 macrophages

The regulation of the immune microenvironment by MSCs depends on the participation of immune cells [4]. To investigate the effect of primed/untreated hADSCs transplantation on the changes of immune cells in colitis and colon fibrosis rats, flow cytometry was used to detect and analyze changes in the proportion of B cells, monocytes, macrophages, and T lymphocytes in the spleen of each group. The colitis rat experiments showed that the proportion of CD45RA^+^ B cells in the spleen of the PBS group was lower than that of the control group, but previous studies have shown that in the inflammatory microenvironment B cells also play a key role in immune regulation, which was verified by our findings [22]. Primed hADSCs significantly increased the proportion of CD45RA^+^ B cells and CD43^+^His48^Int−Lo^ monocytes compared with that in PBS and hADSCs groups. The proportion of CD4^+^ T and CD43^+^ His48^Hi^ neutrophils in the PBS group was obviously higher than that in the control group, and the proportion of these cells decreased significantly after primed hADSCs transplantation but had little effect on CD8^+^ T cells (Fig. S4a). In the gut mucosa, immune cells can be found in organized secondary lymphoid structures, collectively known as gut-associated lymphoid tissue, as well as in intestinal tissue draining mesenteric lymph nodes (mLN), embedded between surface epithelial cells, and within the underlying connective tissue [1]. Research has shown that immune cells in secondary lymph nodes play an important role in the progression of IBD [23]. Therefore, we further analyzed the changes in the proportion of immune cells in the mLN of each group by flow cytometry. Similar to the spleen, the primed group exhibited reduced CD4^+^ T cells and increased CD45RA^+^ B cells (Additional file 1: Fig. S4b).

**Fig. 4:**
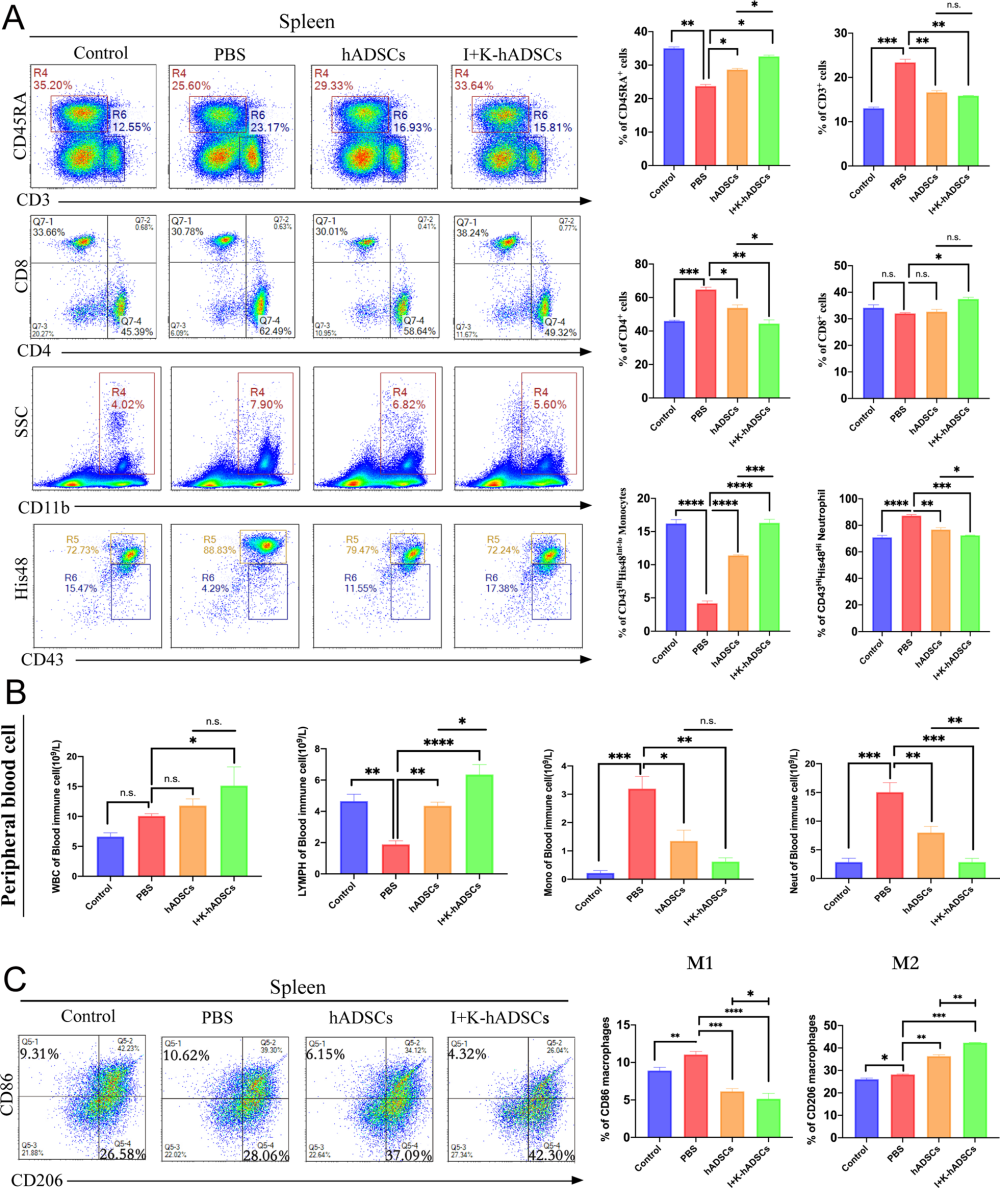
The IFN-γ- and KYNA-primed hADSCs can promoted the polarization of M2 macrophages. (**A**) Rat with the TNBS-induced colon fibrosis were killed on day 46 to harvest the spleen, mLN (*n* = 3). CD45RA^+^ B cells, CD3^+^ T cells, CD4^+^ T cells, CD8^+^ T cells, CD43^+^His48^Int−Lo^ monocytes and CD43^+^His48^Hi^ neutrophils in spleens of each group were detected by flow cytometry and statistical (Int-Lo means interval lower positive cells, Hi means strongly positive cells); (**B**) Blood routine analysis of WBC, lymphocyte, monocytes, and neutrophil in peripheral blood after primed hADSCs treatment (*n* = 3); (**C**) Analysis of macrophage subsets change. The infiltration of CD86^+^M1 macrophages and CD206^+^M2 macrophages in spleens of each group was detected; the following statistical figure shows the analysis of CD86^+^M1 and CD206^+^M2 macrophages in the CD68^+^ cell population (*n* = 3); data are represented as mean ± SEM

We further investigated the immune cell infiltration conditions in a chronic colon fibrosis experiment. Similar to the acute colitis experiment, primed hADSCs, but not untreated hADSCs, treatment significantly increased the proportion of CD45RA^+^ B cells and decreased the number of CD4^+^ T cells and CD43^+^His48^Hi^ neutrophils in the spleen (Fig. 4a). However, primed hADSCs only slightly effect on CD8^+^ T cells (Fig. 4a and Additional file 1: Fig. S4a). Combined with peripheral blood routine results, the number of neutrophils in peripheral blood decreased after primed hADSCs treatment (Fig. 4b). These results suggest that primed hADSCs transplantation can effectively reduce the infiltration of inflammatory cells and inhibit the inflammatory response in injured colon tissue. Monocytes act as precursor cells of macrophages. Differences in the number of monocytes in the peripheral blood and spleen may imply a key role of macrophages in inflammatory response (Fig. 4a and b).

At the early stage of IBD, macrophages infiltrate into the lamina propria and secrete inflammatory factors and chemokines to induce intestinal inflammation, resulting in the damage of epithelial and goblet cells, hyperplasia of ECM, and matrix fibrosis [24]. Currently, it is believed that M1-type macrophages mainly mediate intestinal inflammatory response and fibrosis, while M2-type macrophages have tissue repair and anti-fibrosis effects [25]. To further explore the specific mechanism, we analyzed the spleen and mLN changes of macrophage subtypes after hADSCs transplantation by flow cytometry. Firstly, we observed that the proportion of M1 (CD68^+^ CD86^+^) macrophages in the spleen of the PBS group was significantly higher than that in the control group, indicating that the intestinal inflammatory environment can promote the polarization of macrophages to the M1 type. After primed hADSCs treatment, the M1 macrophages were significantly decreased compared with the hADSCs group. In the primed group, the proportion of M2 (CD68^+^ CD206^+^) macrophages in the spleen was significantly higher than that in the non-pretreated and PBS groups (Fig. 4c). At the same time, in the mLN analysis we also observed similar results that compared with hADSCs group, the primed hADSCs treatment M1 macrophages proportion decreased and M2 macrophages increased (Fig. S4c). We believe that the ability of hADSCs to induce M2 macrophage polarization is the most effective change induced by priming with IFN-γ and KYNA. These results suggest that macrophages play a key role in anti-inflammatory effects and tissue repair.

### hADSCs ameliorated TNBS-induced colitis through an IDO-1-dependent manner

Antifibrotic therapy alone is not effective in the treatment of IBD intestinal fibrosis in clinical, and the preliminary consensus is that antifibrotic therapy alone has a relatively small chance of success if it is not combined with anti-inflammatory therapy. Additionally, if substantial inflammation is confirmed, physicians should initially attempt anti-inflammatory therapy, which might decrease bowel wall edema and thickness, with subsequent relief of the obstructive symptom [26]. Our previous studies have also shown that the anti-inflammatory effect of MSCs is very important in the treatment of liver fibrosis [27]. The above experiments indicated that primed hADSCs of IDO-1 expression increased had significant anti-inflammatory and antifibrotic effects than untreated hADSCs. Therefore, we wanted to observe the effect of deletion of IDO-1 on its therapeutic effect. We demonstrated that the IFN-γ and KYNA priming of hADSCs induced considerable IDO-1 upregulation (Fig. 1c, e and f), and studies have shown that IDO-1 can effectively regulate macrophage polarization [28]. With this, we attempted to determine whether the primed hADSCs promoted M2 macrophage polarization in an IDO1-dependent manner. First, a lentiviral CRISPR vector with IDO-1 gene knockout was constructed (Additional file 1: Fig. S5a). The overlap rate of positively transformed clones was identified by sequencing, and IDO-1 gRNA was successfully connected to the recombinant plasmid vector (Additional file 1: Fig. S5b). hADSCs were transfected with lentivirus followed by purine pressurized screening. Cell morphology was not affected, and there was limited cell death (Additional file 1: Fig. S5c). Western blot analysis confirmed the knockout efficiency, and IDO-1 was not expressed at the protein level after IFN-γ or KYNA treatment (Additional file 1: Fig. S5d).

**Fig. 5:**
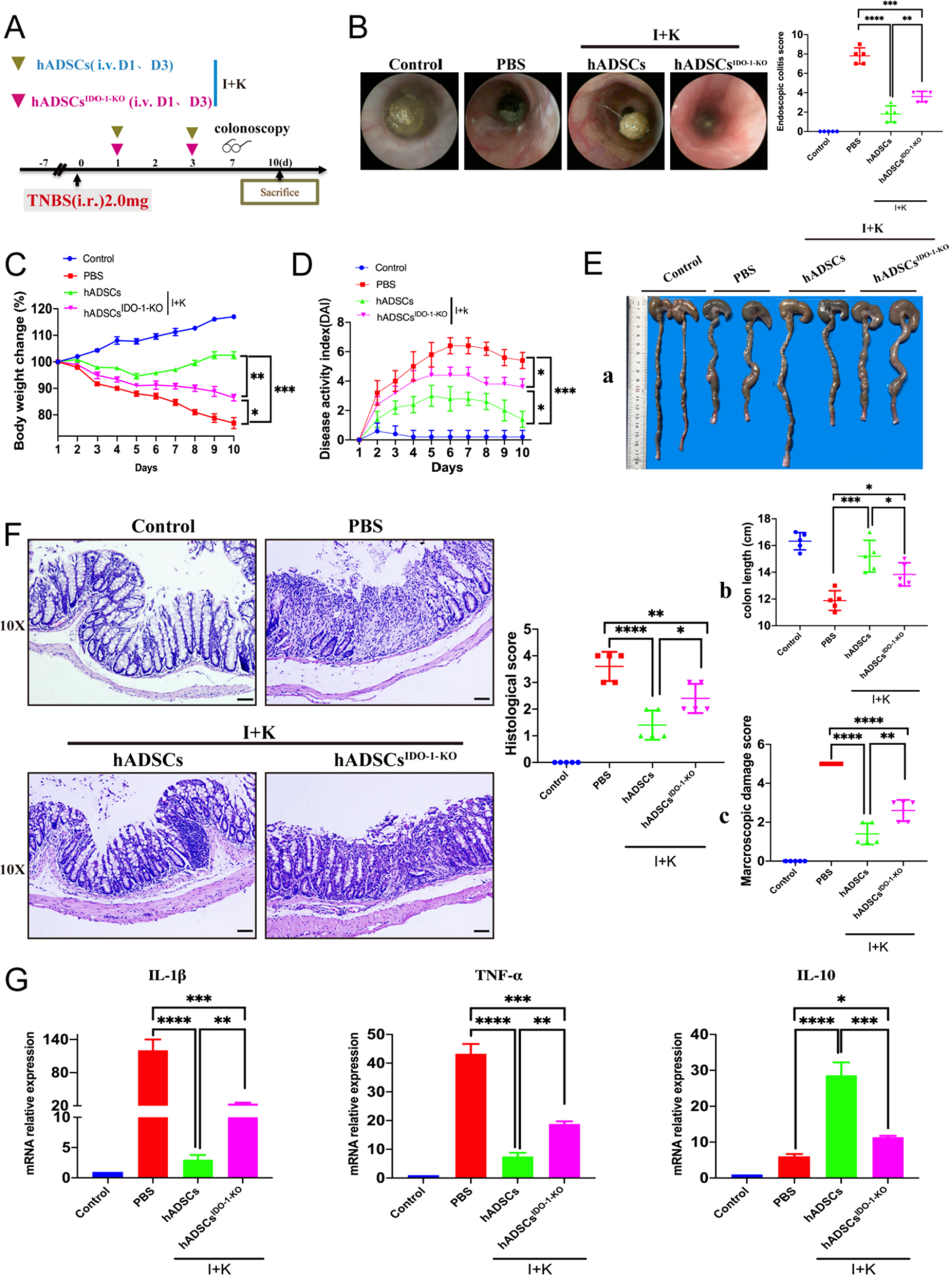
hADSCs ameliorated TNBS-induced colitis through an IDO-1-dependent manner. (**A**) Experimental layout of hADSCs and hADSCs^IDO−1−KO^ transplantation in the TNBS-induced acute colitis in rats; (**B**) Endoscopy observed and statistical on the 7th day (*n* = 5); (**C**) Body weight change (*n* = 5); (**D**) DAI score (*n* = 5); (**E**) Rats were killed at the 10th day, a. anatomic observation (*n* = 5). b. length of the colon was counted (*n* = 5). c. the lesions of the colon were observed (*n* = 5); (**F**) H&E staining was performed to observe the structural integrity of intestinal and the infiltration of immune cells in each group and score (Magnification 10X; Scale bar = 100 μm; *n* = 5); (**G**) qPCR detection mRNA expression changes of inflammatory cytokines IL-1β, TNF-α and anti-inflammatory molecule IL-10 (*n* = 5). Data are represented as mean ± SEM

After obtaining a stable hADSC-IDO-1-KO cell line (hADSC^IDO-1-KO^), we constructed an acute colitis model and performed treatment experiments (Fig. 5a). The results showed that after primed hADSCs^IDO-1-KO^ treatment, rats still had diarrhea and malaise, and colonoscopy revealed that the primed hADSC^IDO-1-KO^ group still had mild diarrhea, intestinal wall congestion, and inflammatory exudation (Fig. 5b, left). The score under endoscopy for primed hADSCs^IDO-1-KO^ was improved compared with the PBS group but could not be compared with primed hADSCs (Fig. 5b, right). We found that primed hADSCs^IDO-1-KO^ reduced body weight loss and improved DAI scores, which were significantly lower than those in the primed hADSCs group (Fig. 5c and d). These results indicate that the treatment effect on colitis was significantly reduced after IDO-1 knockout. Anatomical analysis showed that, compared with the primed hADSCs group, the hADSC^IDO-1-KO^ group had obvious congestion and edema, colon dilation, and colon length that could not be effectively protected (Fig. 5e).

Pathological analysis showed that, compared with the PBS group, the mucosal structure of the colon tissue in the primed hADSC^IDO-1-KO^ group recovered, but epithelial cells and goblet cell damage, gland disorder, and inflammatory cell infiltration were observed compared to the primed hADSCs group (Fig. 5f). Subsequently, we measured the mRNA expression of pro-inflammatory and anti-inflammatory molecules. As shown, primed hADSC^IDO-1-KO^ inhibited the pro-inflammatory molecules TNF-α and IL-1β and increased the anti-inflammatory molecule IL-10, which was significantly decreased compared to the primed hADSCs (Fig. 5g). Overall, IDO-1 was essential for the protective effects of primed hADSCs.

### IFN-γ and KYNA improved hADSCs homing to the colon tissue

**Fig. 6:**
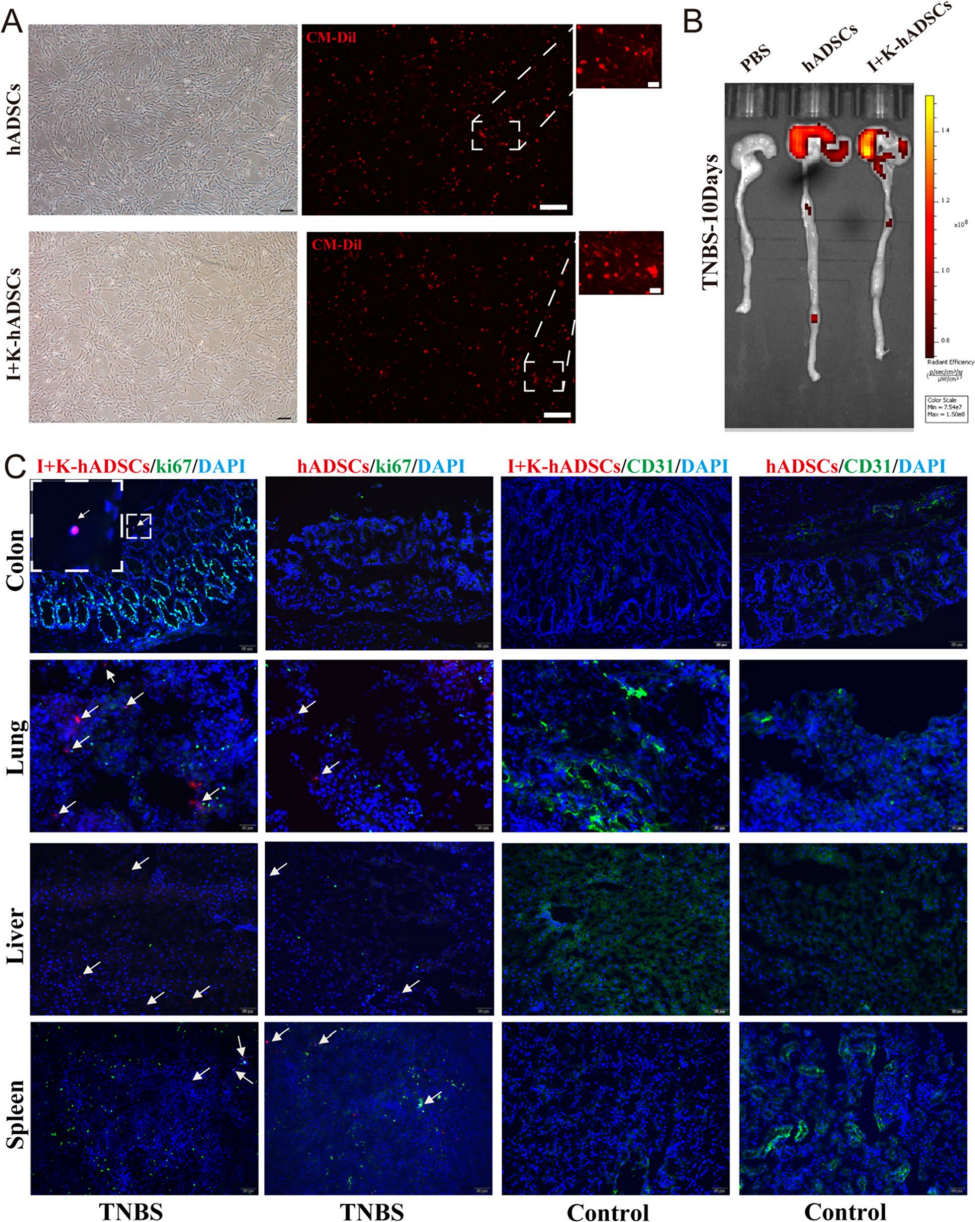
The IFN-γ and KYNA improved hADSCs homing to the colon tissue. (**A**) The morphology of Dil-labeled hADSCs and primed hADSCs was observed under microscope (scale bar, respectively, 50, 100, 10 μm); (**B**) The rats were killed on day 10 and the fluorescence signals of Dil-labeled cells in the colon and small intestine were observed under the IVIS system; (**C**) In vivo distribution of hADSCs and primed hADSCs was observed by immunofluorescence staining of the colon, lung, liver, and spleen. The white arrow points to the Dil-labeled hADSCs (Scale bar, 50 μm)

Next, we investigated whether there was a difference in the ability of the primed/untreated hADSCs to home colon tissue. After injection, the cells were labeled with CM-Dil/DiR for IVIS and section-tracking long-term observations (Additional file 1: Figs. S6a and 6a). The red fluorescent tracer DiR/CM-Dil is a non-specific cell membrane-bound substance, which is widely used for stem cell tracing [29]. First, we used the slice tracking of CM-Dil; rats were killed at 12 h, 24 h, 3 days, and 10 days after cell injection and cryosections were analyzed from multiple organs. Fluorescent signals of Dil-labeled primed and non-pretreated hADSCs were detectable 12 h post-injection, and attenuated signals were still observed 10 days post-injection (Fig. 6b). Dil-labeled cells were predominantly distributed in the lungs, with a small fraction of cells observed in the liver and spleen. To determine the location of hADSCs in the lungs and liver, immunofluorescence staining for the vascular endothelial cell marker CD31 and proliferation marker Ki67 was performed. We observed both primed and non-pretreated hADSCs in the vicinity of endothelial cells in the lungs, suggesting that the cells may be occluded in the pulmonary vessels (Fig. 6c). Dil-labeled primed hADSCs (white arrows) rather than non-pretreated hADSCs were observed in the colon and small intestine after intravenous administration, indicating that IFN-γ and KYNA can promote the migration of hADSCs to inflamed colon tissues (Fig. 6b and c).

The rats were treated with the IVIS live imaging instrument on day 3, 10, and 15, and the migration and distribution of DiR-labeled cells in rats were observed (Additional file 1: Fig. S6b). The untreated and primed hADSCs entered the body, first gathered in the lungs along with the blood circulation, and then gradually distributed in the liver and spleen (Additional file 1: Fig. S6c). The fluorescence signal of primed hADSCs increased on day 10, but the fluorescence signal intensity of hADSCs decreased (Fig. S6d). DiR-labeled primed hADSCs signals were still observed in the abdomen on day 15, but hADSCs decayed so could not be observed. Besides, rats injected with primed and untreated hADSCs were not seen in any organ when not induced with TNBS (Fig. 6c and Additional file 1: S6). The transplanted cells were quickly metabolized or cleared by macrophages after day 10, which also indicates that a series of cytokines and chemokines released from inflammatory and injury sites are key to promoting the homing of primed hADSCs. Therefore, this suggests that untreated and primed hADSCs both had the homing ability to the inflammatory site, but primed hADSCs could migrate to the inflammatory site of the injured intestine more effectively and survive for an extended time, exerting their tissue repair and immune regulatory functions.

**Fig. 7:**
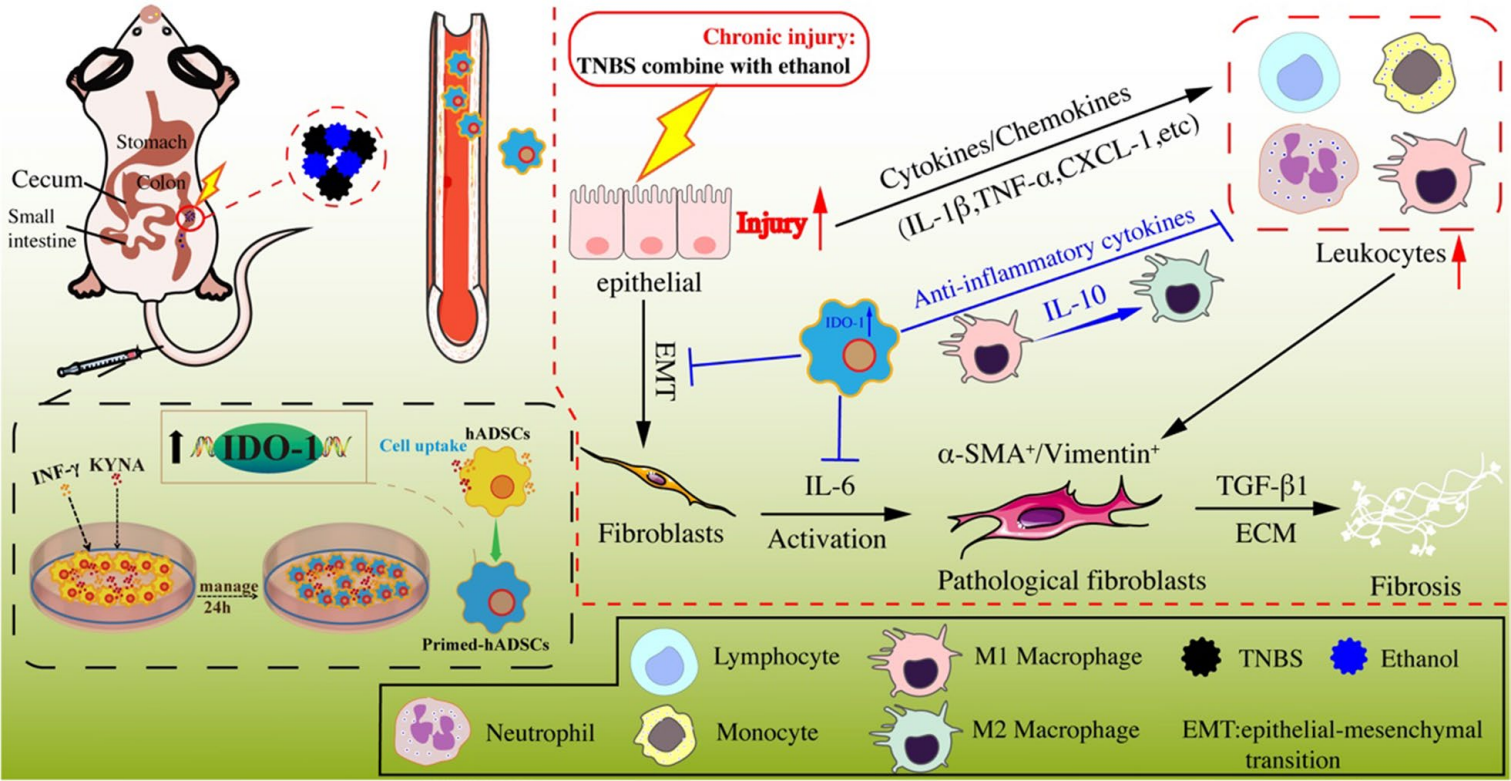
A schematic model of the anti-fibrosis effects of hADSCs pretreated with IFN-γ and KYNA on TNBS-induced CD-like colonic fibrosis. IDO-1 induced by IFN-γ and KYNA catalyzes tryptophan metabolism to produce KYN or KYNA in hADSCs, promotes the polarization of intestinal macrophages to the anti-inflammatory M2, increases the expression of IL-10 to inhibit inflammation, and consequently alleviates IBD and colon fibrosis

Overall, IDO-1 induced by IFN-γ and KYNA catalyzes tryptophan metabolism to produce KYN or KYNA in hADSCs, promotes hADSCs homing and the polarization of intestinal macrophages to the anti-inflammatory M2 and increases the expression of IL-10 to inhibit inflammation, and consequently alleviates colitis and colon fibrosis (Fig. 7).

## Discussion

L-Tryptophan (Trp) is an essential amino acid that is obtained exclusively from dietary intake in humans. Trp and its metabolites have key roles in diverse physiological processes, ranging from cell growth and maintenance, in which Trp serves as a building block of proteins, to the coordination of organismal responses to environmental and dietary cues, in which Trp metabolites serve as neuro-transmitters and signaling molecules [30]. Together, these functions suggest that, during evolution, Trp metabolism has become part of the cellular and organismal communication strategies that align food availability with physiology and behavior. IDO-1, the first enzyme discovered to initiate immunosuppressive kynurenine pathway metabolism. IDO-1 and tryptophan-2,3-dioxygenase (TDO) represent a key intracellular immune checkpoint [31]. Since the discovery of its immunosuppressive effects, a growing body of evidence supports the key role of IDO-1 in immune regulation [32, 33]. Activation of TDO, which catalyzes the same reaction as IDO-1, similarly affects the immune response by inhibiting T cell proliferation, affecting the polarization of macrophages and restraining antitumor immune responses [34, 35]. Although the IDO-1-related enzyme IDO-2 may support IDO-1-mediated immune tolerance, the physiological functions of the IDO-2 enzyme and its roles in disease conditions involve KP activity (Kynurenine (Kyn) pathway (KP), the major pathway in the metabolism of the essential amino acid tryptophan, which contains many immune-active and neuroactive intermediate metabolites) are still unclear [30]. Trp degradation is thought to suppress immune cells through the formation of immunosuppressive Trp catabolites [36, 37] and by Trp depletion [38, 39]. Studies have demonstrated an integral role of IDO-1 in the immunomodulatory capacity of MSCs [10]. This enzyme catalyzes the first and rate-limiting step of tryptophan catabolism along the KP. Notably, IDO-1 and tryptophan metabolites, such as kynurenine, kynurenic acid (KYNA), and 3-hydroxyanthranilic acid, have been documented to modulate the functions of immune cells and regulate the expression of inflammation-associated genes. IDO-1 is expressed at low levels under normal conditions. A variety of inflammatory cytokines, especially IFN-γ, can induce the expression of IDO-1. Studies have found that KYNA can also induce the expression of IDO-1 in vitro [11, 12].

In the tissue regeneration and repair of gastrointestinal disorders, including IBD, MSC transplantation has proven to be one of the most promising therapeutic methods [40]. Many clinical trials based on autologous/allogeneic hADSCs for the treatment of Crohn’s disease perianal fistula have been carried out [41–53], including long-term clinical follow-ups in multiple countries and centers around the world. These studies have observed that hADSCs have good therapeutic efficacy, but the mechanism by which MSCs treat enteritis and repair tissue damage has not been fully elucidated. Previous studies have shown that the immunomodulatory properties of MSCs need to be stimulated by inflammatory factors, such as IFN-γ, TNF-α, and IL-1β [4]^.^ In addition, the signal pathway mediated by Toll-like receptors can also activate the immune-modulatory properties of MSCs [54]. In the present study, we used transcriptome sequencing combined with qPCR and Western blotting to show the immunoregulatory ability of hADSCs pretreated with IFN-γ and KYNA. Subsequent in vivo experiments also proved that primed hADSCs are better than untreated hADSCs in the treatment of Crohn’s disease-like colitis and colonic fibrosis in rats.

A large number of inflammatory factors are present in the microenvironment of colon injury [1]. At present, it is known that TNF-α, IL-1β, and IL-6 are known to be closely related to the progression of enteritis. TNF-α is a key factor in the pathogenesis of IBD [1, 55]. The expression of TNF-α in the inflammatory site of CD patients is significantly increased, which activates monocytes, macrophages, lymphocytes, and neutrophils in the inflammatory microenvironment, which plays a key role in the amplification and prolongation of inflammation [56]. The release of IL-1β in the colon further promotes immune cell infiltration, leading to local neutrophil infiltration and Th17 cell differentiation. IL-6 is secreted mainly by activated macrophages, lymphocytes, and epithelial cells, and its biological effects are similar to those of IL-1β. It can activate intercellular adhesion molecule-1, an adhesion particle that plays a key role in the interaction between neutrophils and intestinal epithelial cells in patients with IBD [57]. The production of IL-6 is thought to be related to the chronic intestinal inflammatory response. Therefore, patients with active CD have significantly higher serum levels of IL-6 than those in healthy adults and patients with CD in remission [58]. In summary, TNF-α, IL-1β, and IL-6 are important inflammatory mediators of colon tissue congestion, edema, and dysfunction. In the CD-like rat model, we found that the expression levels of TNF-α, IL-1β, CXCL-1, and IL-6 in the colon tissue were significantly higher than those in the control group. Compared with untreated hADSCs, primed hADSCs can effectively inhibit the overexpression of these pro-inflammatory factors. Simultaneously, the expression of the anti-inflammatory molecule IL-10 was significantly higher in the primed hADSCs treatment group than in the other groups. Based on the transformation relationship between colitis and intestinal fibrosis, we believe that the prerequisite for controlling fibrosis is to inhibit the inflammatory response. Subsequently, chronic colon fibrosis rats transplanted with primed hADSCs and untreated hADSCs also showed an anti-inflammatory effect similar to that of acute colitis therapeutic experiments.

In a rat model of TNBS-induced chronic colon fibrosis, the colon tissue undergoes long-term inflammatory damage and re-repair process, excessive proliferation, and activation of colonic stromal cells, resulting in a large amount of ECM deposition, excessive accumulation, and insufficient degradation of ECM, causing abnormal intestinal structure and function, ultimately leading to the formation of intestinal wall fibrosis. The main components of the intestinal ECM include type I, II, and IV collagen fibers [59]. In patients with CD, the content of total collagen increases significantly, and a large number of collagen fibers are excessively deposited, separated, and extended between smooth muscle cells, which destroys the structural integrity of the intestinal wall and causes it to thicken [2].

α-SMA has been recognized as a marker protein of myofibroblasts [60]. Under physiological conditions, α-SMA is only found in smooth muscle-derived cells, but under pathological conditions, certain mesenchymal cells such as fibroblasts, which can be activated to express α-SMA, transdifferentiate into myofibroblasts and proliferate to secrete a large amount of ECM. We found that the mRNA and protein levels of α-SMA increased significantly in rats with TNBS-induced colonic fibrosis. After the primed hADSCs transplantation, α-SMA expression and secretion were significantly suppressed. TGF-β1 is a key regulator factor in the process of tissue and organ fibrosis and is the master switch for inducing fibrotic lesions [17]. Favorable evidence supporting this theory is the discovery that TGF-β1 and its receptors are overexpressed in fibroblasts in patients with CD stenosis. In addition, TGF-β1 can stimulate the synthesis of ECM through multiple links, such as promoting fibrogenesis. Cells express α-SMA and inhibit ECM-degrading enzymes (MMPs) and plasminogen protease when collagen synthesized its activity and inhibits the expression of MMP-2 and MMP-9 by stimulating the production of Timp-1. As a result, a large amount of ECM is deposited on the intestinal wall, resulting in intestinal wall fibrosis. We tested the mRNA expression of TGF-β1, MMP-2, MMP-9, and Timp-1 in the colon tissues of each group, which showed that the chronic colon fibrosis group had increased expression of TGF-β1, MMP-2, MMP-9, and Timp-1 increased compared to the control group. After primed hADSCs transplantation, the expression of fibrosis-related genes was significantly inhibited.

EMT means that epithelial cells lose their polarity and transform into fibroblasts [20]. EMT is involved in many pathological processes, including fibrosis of tissues and organs and metastasis of cancer. EMT is not an extreme phenotype of epithelial and mesenchyme, but an intermediate state in which epithelial/stromal cells are mixed [61]. During the EMT process, the most significant change is the disappearance of epithelial cell markers, in which the expression of E-cadherin is significantly reduced. The main function of E-cadherin is to maintain the lateral contact of epithelial cells and maintain cell contact and relative immobility through adhesion connections. Upregulation of vimentin mediates the downregulation of E-cadherin. Vimentin is a cytoskeletal protein that can reduce E-cadherin transport to the cell surface. It is the most common mesenchymal cell marker and main molecular marker of EMT. We detected the changes in the expression of E-cadherin and vimentin by Western blotting and immunofluorescence staining and showed that the TNBS-induced chronic colon fibrosis process is accompanied by a significant EMT process, with a decrease in E-cadherin expression and an increase in vimentin expression. After primed hADSCs treatment, the EMT process related to fibrosis was significantly inhibited.

Macrophages play a key role in repairing tissue damage and the pathogenesis of a variety of human autoimmune diseases [24]. Due of the strong heterogeneity of macrophages, they can differentiate into subgroups of different phenotypes and perform different functions under different environmental stimuli. Th1 cytokines or lipopolysaccharide can stimulate macrophages to differentiate into the M1 type; Th2 secretion of IL-4 and IL-13 can induce macrophages to differentiate into the M2 type. M1 and M2 macrophages are in a dynamic balance during the normal inflammatory response. Under certain conditions, M1 and M2 subtypes can be converted to each other, and deviation to either side leads to different outcomes of inflammation [25]. A number of verified MSCs can polarize M0 or M1 macrophages to the M2 type through their immunomodulatory function and participate in the inhibition of inflammation and tissue repair process [28]. In the present study, we used flow cytometry to detect the proportion of CD68, CD86, and CD206 cell infiltration in the spleen tissue of each rat group. We found that in the primed hADSCs transplant group compared to other groups, the infiltration of CD68^+^ CD86^+^ M1-type macrophages in the spleen tissue was significantly reduced, while the CD68^+^ CD206^+^ M2-type macrophages were significantly increased. We speculate that IFN-γ and KYNA enhance the immune regulatory ability of hADSCs by upregulating IDO-1, increasing autocrine IL-10, and inducing M1 macrophages to polarize to M2 macrophages, which secrete IL-10. This cytokine participates in the immune regulation process of the inflammatory microenvironment, effectively inhibiting the inflammatory response of the intestinal injury site, inhibiting the fibrosis process, and repairing the damaged intestinal tissue.

The mechanism of action of hADSCs also includes directional tropism, migration, and homing ability. Previous studies have found that MSCs can protect against inflammatory damage and the tumor microenvironment to play a regulatory role [62]. The therapeutic effect of MSCs depends on their interaction with the microenvironment and a variety of cells in the body. In the current study, we found that primed hADSCs in vivo can home to the intestinal inflammation injury site for a longer time than untreated hADSCs, existing for more than 10 days.

In conclusion, the present study found that IFN-γ- and KYNA-primed hADSCs have a significant therapeutic effect on acute colitis and chronic colon fibrosis in rats, which ameliorates inflammatory responses and further promotes the functional and structural recovery of colitis through IDO-1-mediated polarization of M2 macrophages. Priming of MSCs with IFN-γ and KYNA is a promising new strategy to improve the therapeutic efficacy of MSCs, which warrants further investigation.

## Conclusions

Collectively, we demonstrated that primed hADSCs pretreated with IFN-γ and KYNA in vitro could regulate the polarization of M1 macrophages to M2 macrophages in colon injury fibrosis rats through the upregulation of IDO-1 and increase the production of IL-10. By regulating the activity and proliferation of myofibroblasts, inhibiting the accumulation of ECM and the over-activation of the EMT process, primed hADSCs can effectively inhibit the deposition of intestinal fibrin and fibrosis. By enhancing the homing ability of hADSCs to the site of inflammatory injury, it can better play the role of inflammation regulation and tissue repair (Fig. 7). Those findings will contribute to the development of effective treatment for IBD.

## Supplementary Information

**Additional file1 :**

## Data Availability

The datasets used and/or analyzed during the current study are available from the corresponding author on reasonable request.

## Abbreviations

MSCs: Mesenchymal stem cells
hADSCs: Human adipose-derived MSCs
KYNA: Kynurenic acid
primed hADSCs: HADSCs with IFN-γ and KYNA pretreatment
IBD: Inflammatory bowel disease
CD: Crohn’s disease
UC: Ulcerative colitis
IDO-1: Indoleamine 2,3-dioxygenase-1
COX2: Cyclooxygenase-2
GAPDH: Glyceraldehyde-3-phosphate dehydrogenase
TNBS: 2,4,6-Trinitrobenzen sulfonic acid
DAI: Disease activity index
ECM: Extracellular matrix
EMT: Epithelial–mesenchymal transition
MPO: Myeloperoxidase
E-cad: E-cadherin
α-SMA: Alpha smooth muscle actin
IL-1β, 6, and 10: Interleukin-1 beta, 6, and 10
IFN-γ: Interferon-γ
TNF-α: Tumor necrosis factor-α
Acta2: Actin alpha 2
Timp1: Tissue inhibitor of metalloproteinases 1
WB: Western blot
