# Supplementary material for: Therapeutic efficacy of human adipose mesenchymal stem cells in Crohn’s colon fibrosis is improved by IFN-γ and kynurenic acid priming through indoleamine 2,3-dioxygenase-1 signaling
Source: Stem Cell Res Ther. 2022 Sep 8;13:465. doi: 10.1186/s13287-022-03157-8 (PMC9461110; doi:10.1186/s13287-022-03157-8)
Supplement: Supplementary file 1 — Additional file1 : [file 13287_2022_3157_MOESM1_ESM.docx]

**Supplementary Table S1. IDO-1 sgRNA primer sequence**

| Gene Name | Sequence (5’to 3’) |
| --- | --- |
| gIDO-1-F | caccGTACCATATTGATGAAGAAGT |
| gIDO-1-R | aaacACTTCTTCATCAATATGGTAC |

**Supplementary Table S2. TNBS/ethanol solution preparation**

1. 2%TNBS/ethanol solution

| Reagent | Dosage |
| --- | --- |
| TNBS(5%W/V) | 400μL |
| Ethanol absolute | 400μL |
| Normal saline | 200μL |

1. 1.5%TNBS/ethanol solution

| Reagent | Dosage |
| --- | --- |
| TNBS(5%W/V) | 300μL |
| Ethanol absolute | 400μL |
| Normal saline | 300μL |

1. 1.0%TNBS/ethanol solution

| Reagent | Dosage |
| --- | --- |
| TNBS(5%W/V) | 200μL |
| Ethanol absolute | 400μL |
| Normal saline | 400μL |

**Supplementary Table S3. Antibodies for used**

| Antibodies | Source | Identifier |
| --- | --- | --- |
| PE anti-human CD73 Antibody | Biolegend | Cat#344004 |
| PE anti-human CD90 (Thy1) Antibody | Biolegend | Cat#328110 |
| PE anti-human CD44 Antibody | Biolegend | Cat#338808 |
| PE anti-human CD105 Antibody | Biolegend | Cat#323206 |
| PE anti-human CD29 Antibody | Biolegend | Cat#303004 |
| PE anti-human CD45 Antibody | Biolegend | Cat#304008 |
| PE anti-human CD11b Antibody | Biolegend | Cat#301306 |
| FITC anti-human CD34 Antibody | Biolegend | Cat#343504 |
| PE anti-human HLA-DR Antibody | Biolegend | Cat#307606 |
| PE anti-human CD14 Antibody | Biolegend | Cat#325605 |
| PE Mouse IgG1κ Isotype Ctrl Antibody | Biolegend | Cat#400112 |
| FITC Mouse IgG1κ Isotype Ctrl Antibody | Biolegend | Cat#400109 |
| PE Mouse IgG2a κ Isotype Ctrl Antibody | Biolegend | Cat#400212 |
| PE/Cyanine7 anti-rat CD45RA Antibody | Biolegend | Cat#202315 |
| FITC anti-rat CD4 (domain 2) Antibody | Biolegend | Cat#203305 |
| APC anti-rat CD3 Antibody | Biolegend | Cat#201413 |
| PE/Cyanine7 anti-rat CD11b/c Antibody | Biolegend | Cat#201817 |
| APC/Fire™ 750 anti-rat CD45 Antibody | Biolegend | Cat#202222 |
| PE anti-rat CD8a Antibody | Biolegend | Cat#201705 |
| Alexa Fluor® 647 anti-rat CD43 | Biolegend | Cat#202810 |
| Granulocyte Marker Monoclonal（HIS48） | Thermo | Cat#11057082 |
| Goat anti-Rabbit IgG Secondary | Thermo | Cat#A-11034 |
| Alexa Fluor 555 Goat anti-Rabbit IgG | Thermo | Cat#A-21428 |
| Alexa Fluor 488 Goat anti-Rabbit IgG | Thermo | Cat#A-11034 |
| Alexa Fluor 488 Goat anti-Mouse IgG | Thermo | Cat#A-11001 |
| Alexa Fluor 488 Goat anti-Chicken IgY | Thermo | Cat#A-11039 |
| Alexa Fluor 568 Goat anti-Mouse IgG | Thermo | Cat#A-11004 |
| Alexa Fluor 647 Goat anti-Rat IgG | Thermo | Cat#A-21247 |
| MRC1 Antibody (CD206) | Proteintech | Cat#187041-AP |
| Rabbit Anti-Human IDO Antibody | Proteintech | Cat#132681-AP |
| INOS Polyclonal Antibody | Proteintech | Cat#189851-AP |
| COX2/ PTGS2 Polyclonal Antibody | Proteintech | Cat#123751-AP |
| Anti-E Cadherin antibody | Abcam | Cat#ab76055 |
| Rabbit antibody to Collagen IV | Abcam | Cat#ab6586 |
| Rabbit antibody to α-SMA | Abcam | Cat#ab5694 |
| Rabbit antibody to Laminin | Abcam | Cat#ab11575 |
| Rabbit antibody to Vimentin | CST | Cat#5741 |
| Mouse antibody to PCNA | CST | Cat#2586 |
| Rat antibody to CD45 | CST | Cat#55307 |
| Mouse anti Rat CD68 Antibody | Bio-Rad | Cat#341A647 |
| PE Mouse Anti-Rat CD86 Antibody | BD | Cat#551396 |

**Supplementary Table S4. Quantitative RT-PCR primer sequences of rat**

| Gene name | Sequence (5’to 3’) |
| --- | --- |
| Rat-β-actin-F | CTAAGGCCAACCGTGAAAAGATG |
| Rat-β-actin-R | TACGACCAGAGGCATACAGG |
| Rat-IL10-F | GGGAGAGAAGCTGAAGACCC |
| Rat-IL10-R | TTGAGTGTCACGTAGGCTTCT |
| Rat-IL1β-F | AGGCTGACAGACCCCAAAAG |
| Rat-IL1β-R | CTCCACGGGCAAGACATAGG |
| Rat-TNF-α-F | GATCGGTCCCAACAAGGAGG |
| Rat-TNF-α-R | TTTGCTACGACGTGGGCTAC |
| Rat-IL6-F | TGCCTTCTTGGGACTGATGT |
| Rat-IL6-R | TGGTCTGTTGTGGGTGGTATC |
| Rat-Cxcl1/Groα-F | CCCAAACCGAAGTCATAGCCA |
| Rat-Cxcl1/Groα-R | TTACTTGGGGACACCCTTTAGC |
| Rat-Cxcl2/MIP-2-F | ATGCTGTACTGGTCCTGCTC |
| Rat-Cxcl2/MIP-2-R | GTAGGGTCGTCAGGCATTGA |
| Rat-Acta2-F | CATCACCAACTGGGACGACA |
| Rat-Acta2-R | TCCGTTAGCAAGGTCGGATG |
| Rat-TGF-beta-F | TGGCCAGATCCTGTCCAAAC |
| Rat-TGF-beta-R | GTTGTACAAAGCGAGCACCG |
| Rat-Timp1-F | GGCATAATCTGAGCCCTGCT |
| Rat-Timp1-R | GGGATGGCTGAACAGGGAAA |
| Rat-Mmp2-F | TGGCACCACCGAGGATTATG |
| Rat-Mmp2-R | TGCCCAGAAAAGTGAAGGGG |
| Rat-Mmp9-F | TCGGATGGTTATCGCTGGTG |
| Rat-Mmp9-R | AAGACGCACATCTCTCCTGC |

**Supplementary Table S5. Quantitative RT-PCR primer sequences of human**

| Gene name | Sequence (5’to 3’) |
| --- | --- |
| Human-β-actin-F | CATGTACGTTGCTATCCAGGC |
| Human-β-actin-R | CTCCTTAATGTCACGCACGAT |
| Human-IDO-1-F | TTGCTAAAGGCGCTGTTGGA |
| Human-IDO-1-R | GTCTGATAGCTGGGGGTTGC |
| Human-iNOS-F | TGAACTACGTCCTGTCCCCT |
| Human-iNOS-R | CTCTTCTCTTGGGTCTCCGC |
| Human-PTGS2/Cox2-F | AATCCTTGCTGTTCCCACCC |
| Human-PTGS2/Cox2-R | GTCCGGGTACAATCGCACTT |


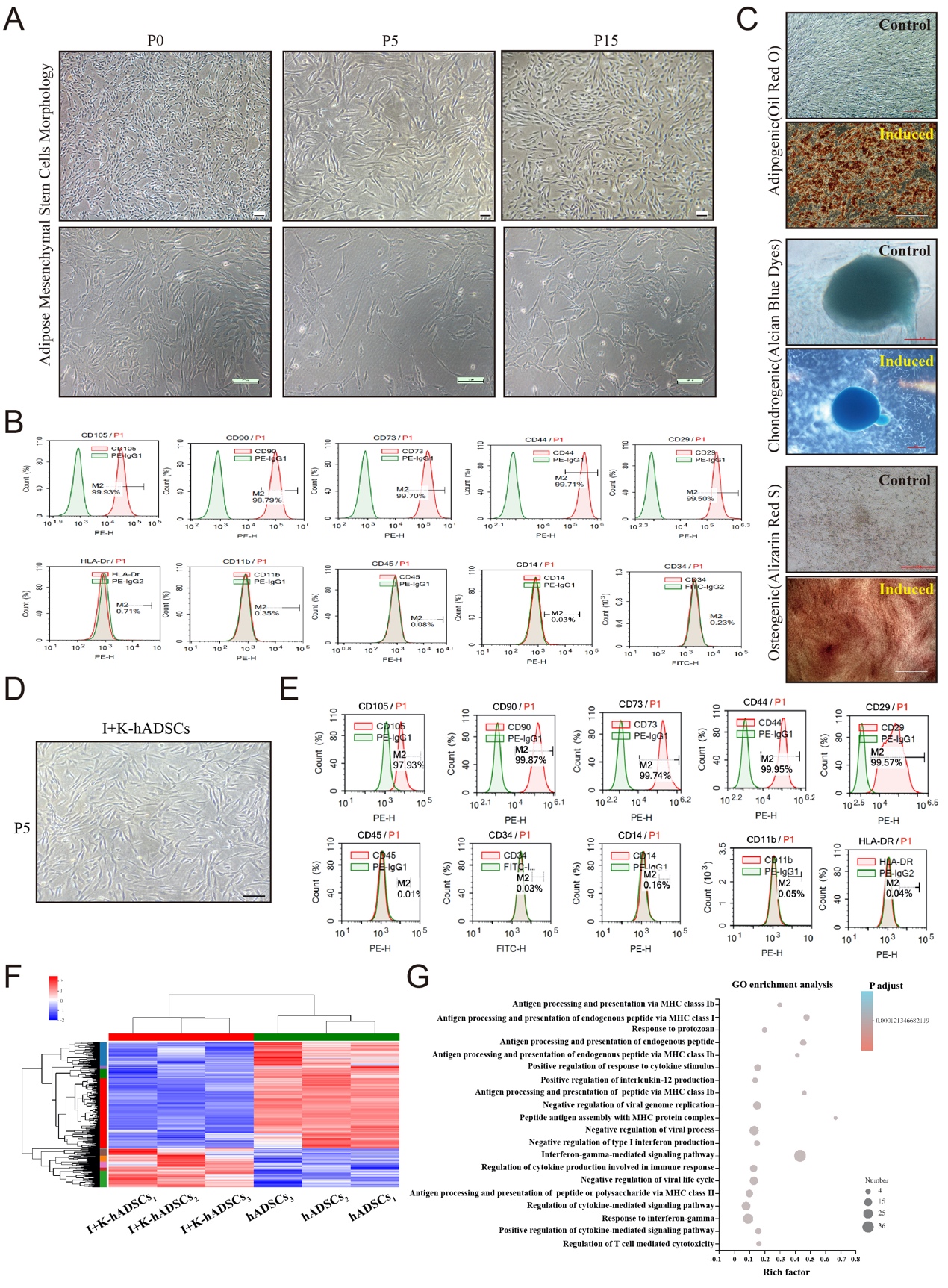


Supplementary Figure 1. **Characteristics of hADSCs and IFN-γ with KYNA primed-hADSCs**. (A) the hADSCs morphology of the P0, the P5 and the P15(scale bars,100μm). Below is a high magnification image (scale bars,100μm). (B) Flow cytometry analysis of surface markers of hADSCs, positive surface marker molecules include (CD105, CD90, CD73, CD44, and CD29); negative surface marker molecules include (HLA-DR, CD11b, CD45, CD14, and CD34). (C) respectively showed adipocytes, chondrocytes and osteoblasts induced by hADSCs and their negative controls (Scale bars, 50μm). (D) the P5 hADSCs morphology with IFN-γ and KYNA pretreated (I+K-hADSCs, Scale bars,100μm). (E) Flow cytometry analysis of surface markers of I+K-hADSCs. (F) Cluster analysis of hADSCs and I+K-hADSCs. (G) GO enrichment analysis of hADSCs and I+K-hADSCs.


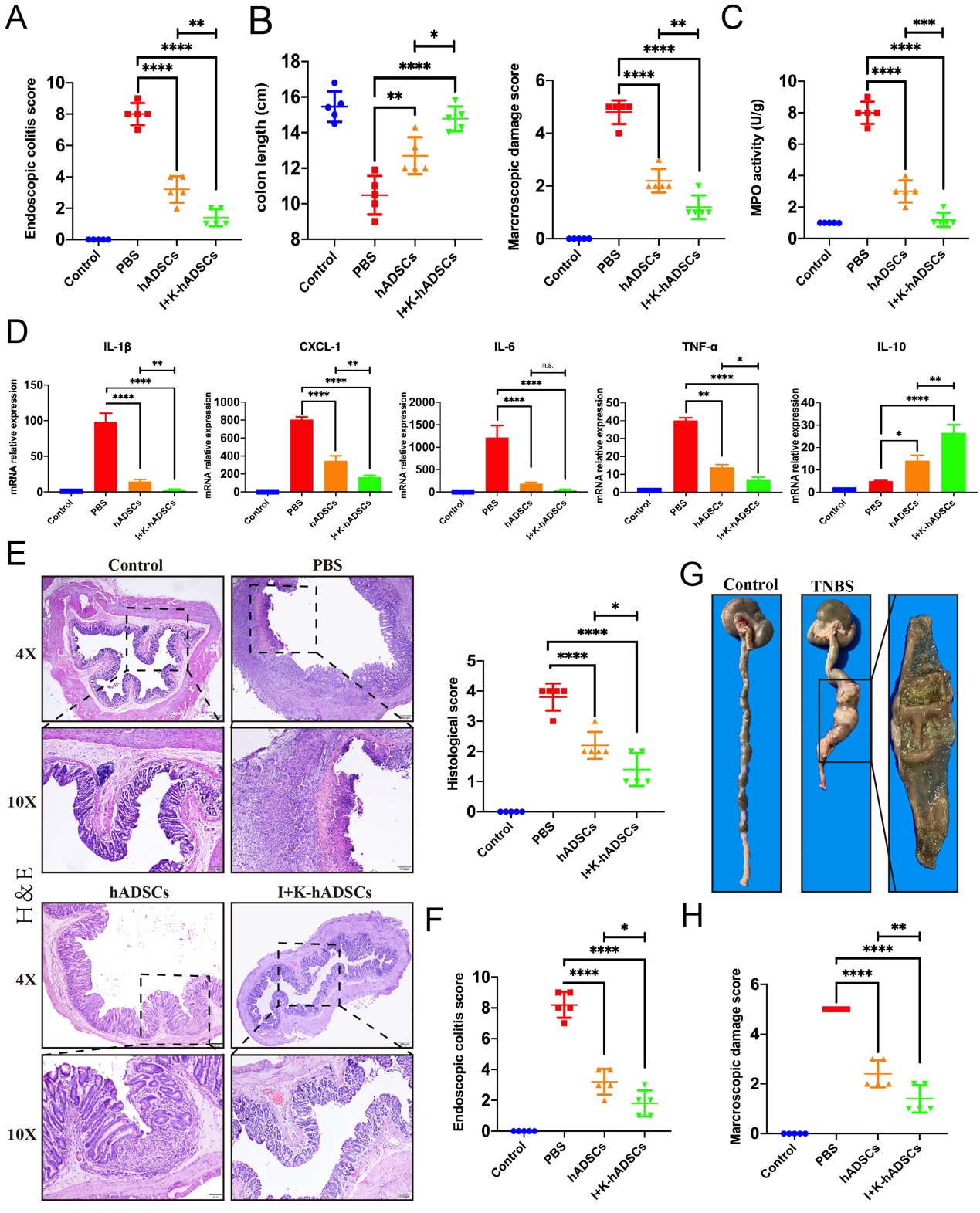


Supplementary Figure 2. **The IFN-γ and KYNA-primed hADSCs than untreated hADSCs more effective ameliorate inflammatory and tissue injured.** (A) Therapeutic efficacy evaluation of acute TNBS model. Endoscopy observed statistical on the 7th day (n=5); (B) Rats were killed at the 10th day, the length of the colon was counted and the lesions of the colon were observed (n=5); (C) MPO activity detected (n=5); (D) Q-PCR detection mRNA expression changes of inflammatory cytokines IL-1β, CXCL-1, IL-6, TNF-α and anti-inflammatory molecule IL-10 (n=5). (E) H&E staining was performed to observe the structural integrity of intestinal and the infiltration of immune cells in each group and score (Magnification 4X and 10X; Scale bar=200, 100 μm; n=5); (F) Endoscopy observed statistical on the 46th day (n=5); (G) Rats were killed at the 46th day, colon fibrosis rat after dissection showed that the colon was shortened, twisted, stiff and deformed, adhered to the surrounding tissues, penetrating ulcer with stool retention in the obvious area of colon dilation and the lesion was covered with pseudo-membrane (amplification); (H) colon injured score after primed and untreated hADSCs treatment (n=5);


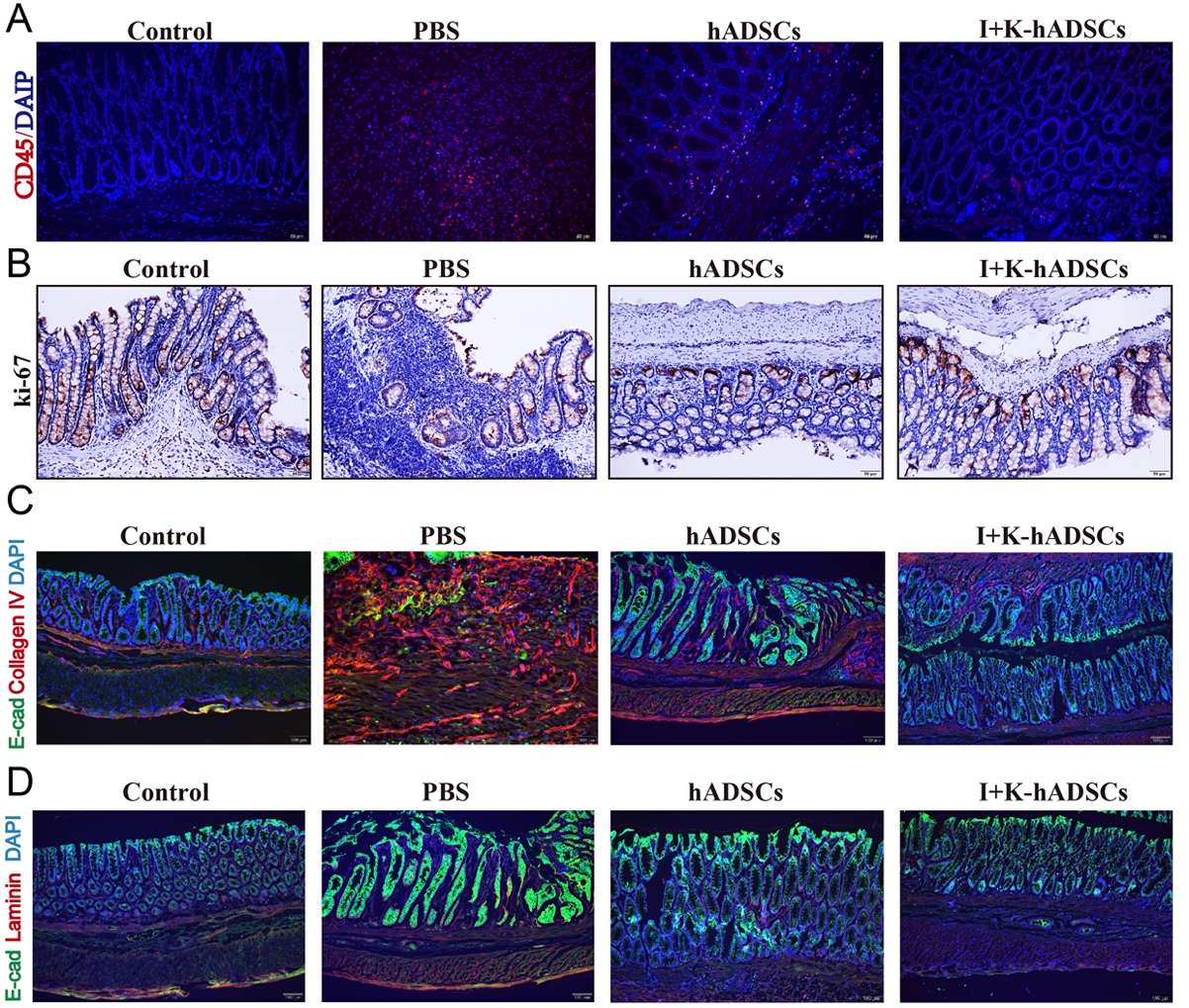


Supplementary Figure 3. **The IFN-γ and KYNA-primed hADSCs than untreated hADSCs more effective inhibited the ECM deposition.** (A) CD45 immunofluorescence markers immune cell infiltration in the intestine (scale bars, 50 μm); (B) The repair ability of epithelial injury was detected by immunochemistry of Ki67 (scale bars, 50 μm); (C) Immunofluorescence staining of the epithelial marker E-cad and collagen marker Collagen IV in frozen sections of the colon (Scale bar, 100 μm); (D) Immunofluorescence staining of the epithelial marker E-cad and stromal marker Laminin in frozen sections of the colon (Scale bar, 100 μm);


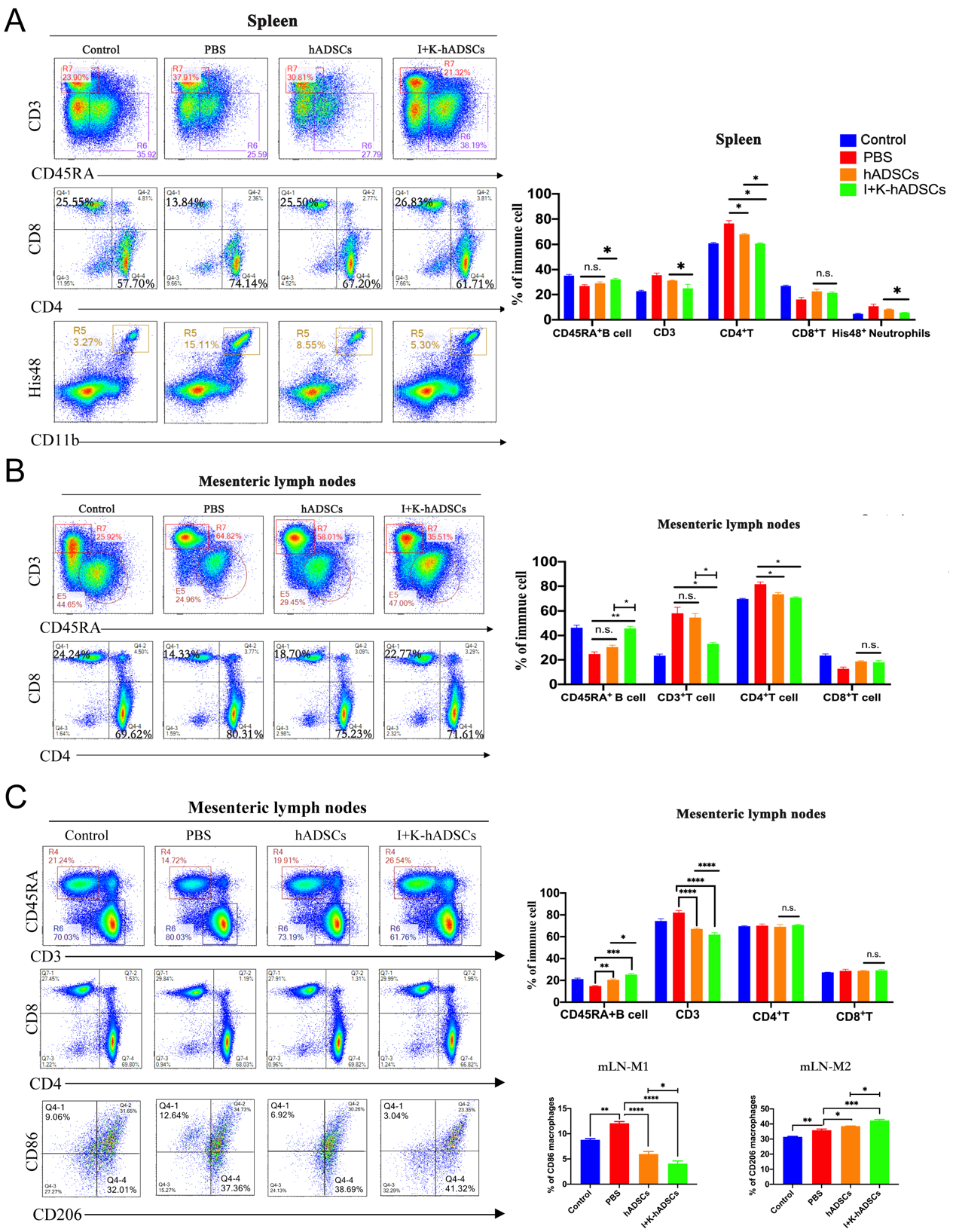


Supplementary Figure 4. **The IFN-γ and KYNA-primed hADSCs than untreated hADSCs more effective inhibited immune cells infiltration.** (A-B) Rat with the TNBS-induced acute colitis were sacrificed on day 10 to harvest the spleen, mLN (n= 3). CD45RA^+^ B cells, CD3^+^ T cells, CD4^+^ T cells, CD8^+^ T cells and CD11b^+^His48^+^ Neutrophils in spleens of each group were detected by flow cytometry and statistical; (C) Flow cytometry detection of mLN in the treatment group of chronic intestinal fibrosis (n= 3).


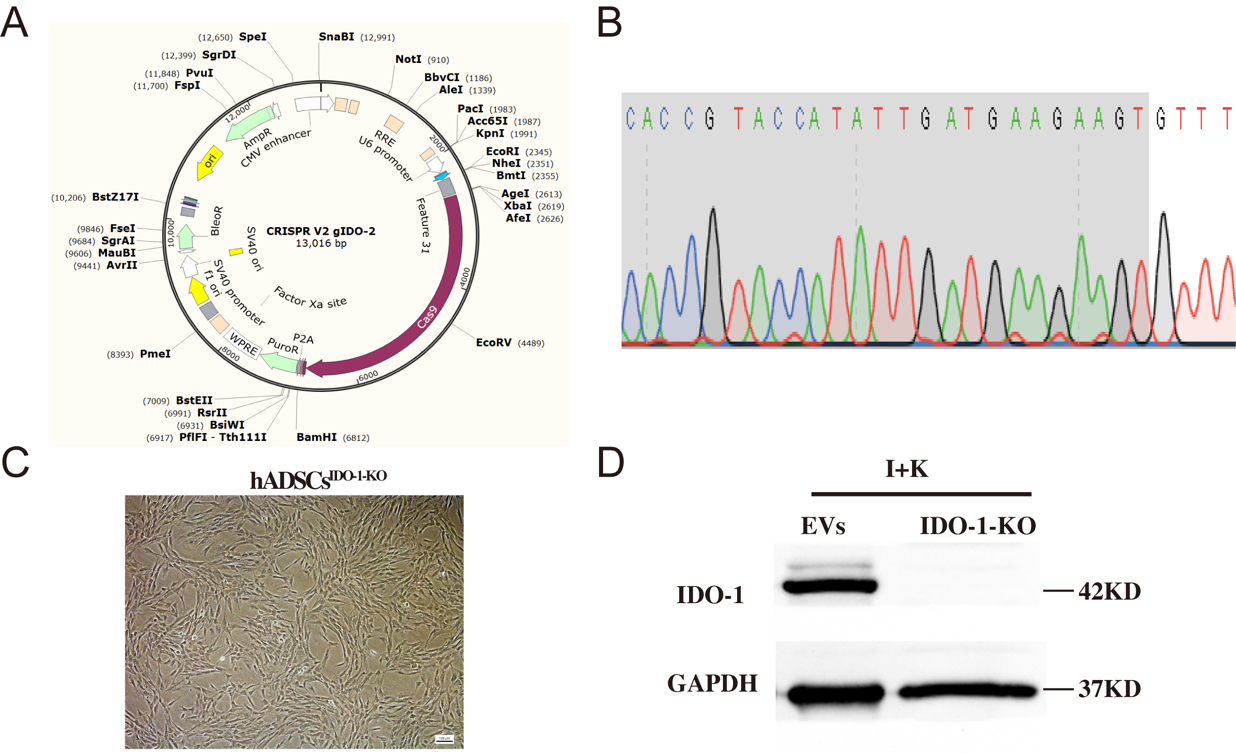


Supplementary Figure 5. **IDO-1 of hADSCs knockout cell lines were constructed**. (A) Schematic diagram of IDO-1-gRNA Lentiviral-CRISPR vector; (B) Sequence diagram of overlap rate of positive clones; (C). hADSCs^IDO-1-KO^ purine screening mirror image (Scale bars, 100 μm); (D) Western blot detected the protein expression of IDO-1.


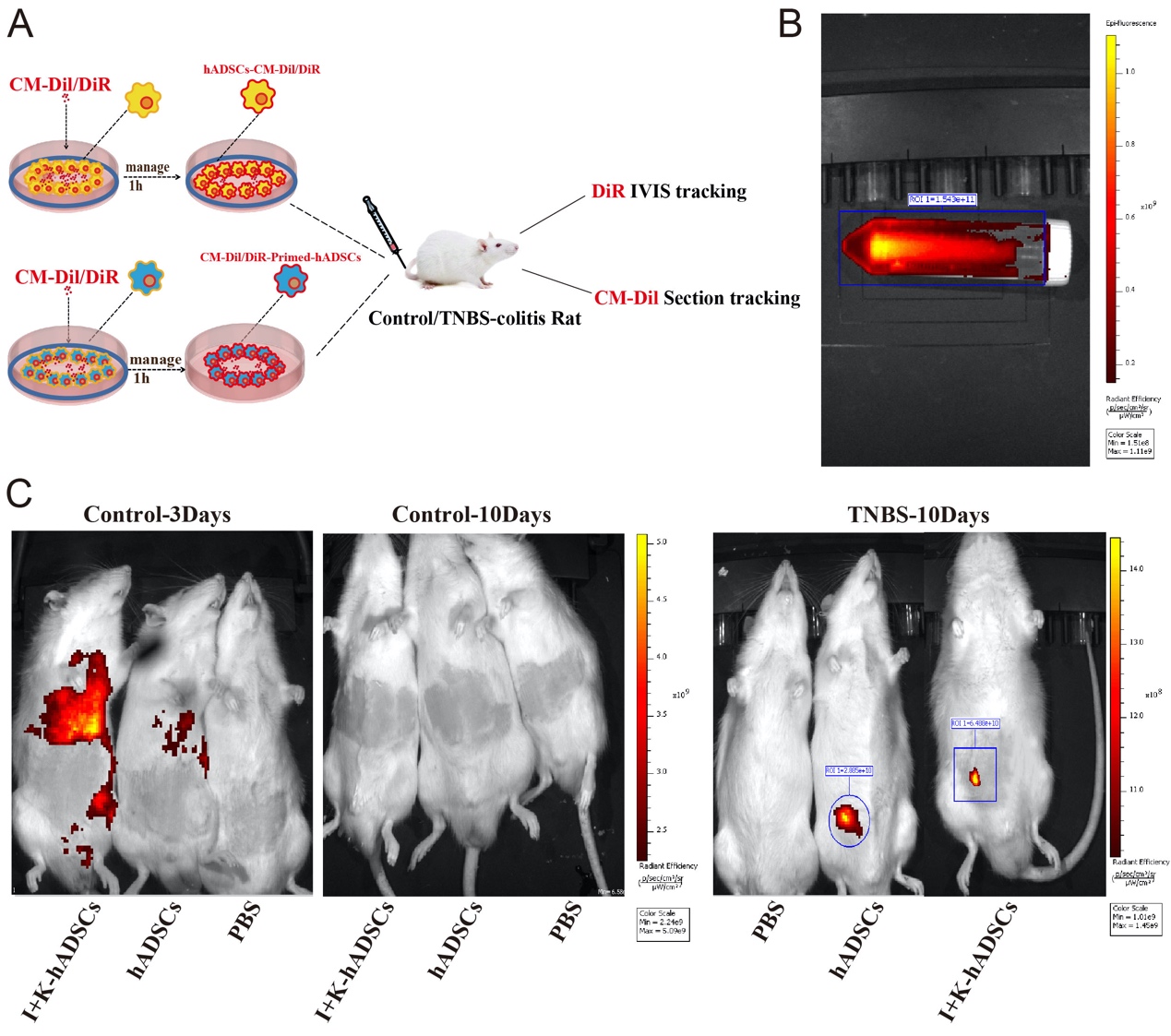


Supplementary Figure 6. **Detection of CM-Dil-labeled hADSCs/primed-hADSCsin rat model.** (A) pattern image of track primed/untreated after injection, cells were labeled with CM-Dil/DiR for IVIS and section tracking long-term observation; (B) IVIS system used to detect fluorescence intensity after cells were labeled with DiR membrane dye; (C) The fluorescence signal intensity of the control group and the TNBS-induced group was measured on day 3 and 10, respectively.
